# Mutation of SPINOPHILIN (PPP1R9B) found in human tumors promotes the tumorigenic and stemness properties of cells

**DOI:** 10.7150/thno.53572

**Published:** 2021-01-19

**Authors:** Eva M Verdugo-Sivianes, Ana M Rojas, Sandra Muñoz-Galván, Daniel Otero-Albiol, Amancio Carnero

**Affiliations:** 1 Instituto de Biomedicina de Sevilla, IBIS, Hospital Universitario Virgen del Rocio, Consejo Superior de Investigaciones Científicas, Universidad de Sevilla, Avda. Manuel Siurot s/n, 41013 Seville, Spain.; 2 CIBERONC, Instituto de Salud Carlos III, 28029 Madrid, Spain.; 3 Centro Andaluz de Biología del Desarrollo (CABD), CSIC-Universidad Pablo de Olavide, Sevilla, Spain.

**Keywords:** SPINOPHILIN, PP1, cancer stem cell, tumorigenesis, stem cell phenotype, pRB, pocket proteins

## Abstract

**Rationale:** SPINOPHILIN (SPN, PPP1R9B) is an important tumor suppressor involved in the progression and malignancy of different tumors depending on its association with protein phosphatase 1 (PP1) and the ability of the PP1-SPN holoenzyme to dephosphorylate retinoblastoma (pRB).

**Methods:** We performed a mutational analysis of SPN in human tumors, focusing on the region of interaction with PP1 and pRB. We explored the effect of the SPN-A566V mutation in an immortalized non-tumorigenic cell line of epithelial breast tissue, MCF10A, and in two different p53-mutated breast cancer cells lines, T47D and MDA-MB-468.

**Results:** We characterized an oncogenic mutation of SPN found in human tumor samples, SPN-A566V, that affects both the SPN-PP1 interaction and its phosphatase activity. The SPN-A566V mutation does not affect the interaction of the PP1-SPN holoenzyme with pocket proteins pRB, p107 and p130, but it affects its ability to dephosphorylate them during G0/G1 and G1, indicating that the PP1-SPN holoenzyme regulates cell cycle progression. SPN-A566V also promoted stemness, establishing a connection between the cell cycle and stem cell biology via pocket proteins and PP1-SPN regulation. However, only cells with both SPN-A566V and mutant p53 have increased tumorigenic and stemness properties.

**Conclusions:** SPN-A566V, or other equivalent mutations, could be late events that promote tumor progression by increasing the CSC pool and, eventually, the malignant behavior of the tumor.

## Introduction

SPINOPHILIN (SPN, also known as PPP1R9B or NEURABIN-2) is a multifunctional protein that acts as a scaffold regulating protein-protein interactions. Indeed, more than 30 partners of SPN have been identified so far 1. SPN is one of the regulatory subunits of protein phosphatase 1 (PP1), with an important role in the dephosphorylation of retinoblastoma protein (pRB) during the cell cycle 1-4. Previous studies demonstrated that loss of SPN induces a proliferative response that reduces the levels of PP1α and increases those of inactive phosphorylated pRB, P-pRB, thus activating the cell cycle 2,5.

The SPN gene is located at 17q21.33, a region frequently associated with microsatellite instability, loss of heterozygosity (LOH) and a high density of well-known tumor suppressor genes, such as BRCA1 5-11. Spn knock-out mice showed reduced lifespan, higher number of spontaneous tumors and increased cellular proliferation in some tissues, such as mammary ducts. Indeed, the combined loss of Spn and p53 activity increased preneoplastic and neoplastic lesions in mammary glands. The loss of Spn increases the response of p53 similarly to cellular senescence induced by oncogenic stress. Thus, once p53 is lost, the loss of Spn increases tumor aggressiveness 3,5.

SPN has been described as a tumor suppressor gene in different human tumors, with low expression levels correlating with worse prognosis 5,12-15. In addition, approximately 20% of lung tumors show loss of SPN, while 38% show low levels, and this decrease is associated with a higher grade of malignancy and mutations in p53, thus confirming their functional relationship 12,16. There is also a positive correlation between the decrease in SPN expression and low levels of the three catalytic subunits of PP1, and this combination is associated with a worse prognosis in squamous cell lung cancer 16.

In breast cancer, SPN plays an important role as a tumor suppressor gene. SPN levels are reduced or lost in approximately 15% of breast tumors, which correlates with higher histological grade, less differentiated phenotype and worse survival. In addition, ER-negative tumors and triple-negative tumors have lower levels of SPN than luminal tumors 17,18. In fact, both SPN and p53 are lost in triple-negative tumors, and this combined loss makes tumors more aggressive 19. The downregulation of SPN in breast cancer cell lines increases the tumorigenic properties and cancer stem cell properties, such as the formation of tumorspheres and the expression of stem cell genes (NANOG, OCT4, SOX2 and KLF4), whereas the overexpression of SPN causes the opposite effect 17,18. Moreover, tumors or cell lines with low levels of SPN showed an enrichment of CD44+ CD24- cells 17, which have been proposed to be cancer-initiating cells in breast tumors 20,21. Therefore, the fact that the loss of SPN causes an increase in the stem cell phenotype could explain why tumors with low SPN levels have a worse prognosis since poor response to chemotherapy and relapse are associated with a greater number of cancer stem cells (CSCs) 15,17,22,23.

PP1 regulatory proteins direct it towards specific substrates to perform specific functions 24-27. The mechanism through which PP1 dephosphorylates pRB is not well understood, and SPN is thought to be the regulatory protein involved in this process 28. The interaction of SPN with PP1 occurs through amino acids 417-583, which includes the PP1-binding domain and the PDZ domain 25. The study of PP1 regulatory proteins involved in the cell cycle is essential since mutations that prevent binding to PP1 or pRB will promote phosphorylation of pRB and eventually cell transformation. Therefore, mutations in SPN in the region of interaction with PP1 might affect and promote the onset and progression of tumorigenesis 1-4,16,17.

In this work, we performed a mutational analysis of SPN in human tumors, focusing on the region of interaction with PP1 and pRB. We characterized an oncogenic mutation of SPN located in the PDZ domain since cells that overexpress SPN-A566V presented a clear increase in some tumorigenic and cancer stem cell properties. The SPN-A566V mutation affects both the interaction between SPN and PP1 and the phosphatase activity of the holoenzyme, especially over the pocket proteins pRB, p107 and p130. Therefore, SPN-A566V, or other equivalent mutations, could be late events that promote tumor progression by increasing the CSC pool and, eventually, the malignant behavior of the tumor.

## Methods

### Mutational analysis

For the mutational analysis, we used human tumor cell lines and tumor samples from the biobank of HUVR-IBIS (Seville, Spain). Total RNA was extracted and purified using the mirVana miRNA Isolation kit (Ambion, Life Technologies), and reverse transcription was performed with 500 ng of mRNA using the High Capacity cDNA Reverse Transcription kit (Life Technologies) according to the manufacturer's instructions. For the reverse transcription, we used a specific primer (CCCCAGTAGCCTTCCAGTTT). We amplified the region of interest by PCR using a MyTaq DNA Polymerase kit (BIOLINE). The PCR mixture (50 µL) contained 5 µL of the reverse transcriptase reaction product, 12.5 µL of 5X MyTaq buffer, 1 µL of 10 µM forward primer (GTTCTCCTCCACACTCTGCT), 1 µL of 10 µM reverse primer (TTCTCGGAGGCGGACTTG), 1 µL of DMSO, 0.5 µL of MyTaq DNA polymerase and 0.2 µL of Pfu DNA polymerase (Promega). The PCR was performed under the following conditions: 3 min at 95 ºC, 40 cycles of 30 s at 95 ºC, 45 s at 62 ºC and 1 min and 30 s at 72 ºC and 3 min at 72 ºC. Finally, the samples were sequenced. The sequencing reaction was performed using the BigDye® Terminator v3.1 Ready Reaction Mix kit (Applied Biosystems) using a forward (GGAGCTCCTTGAACTTGTGC) and a reverse (GGAGGAGGACGACGAAGAC) primer. PERFORMA® V3 96-Well Short Plate purification plates (EdgeBio) were used, and the sequencing was performed on an automatic 3500 8-capillary sequencer (Applied Biosystems).

### Site-directed mutagenesis

For the generation of the mutation, a 1.8-kb fragment of the pCMV6-SPN (OriGene RC213696) was cloned into a pBluescript SK(-) plasmid. The mutagenic PCR mixture (50 µL) contained 150 ng of plasmid DNA, 10 µL of 5X Q5 reaction buffer, 1 μL of 10 mM dNTPs, 2.5 µL of 10 µM forward primer (GAGCTTCGCGGTGTCTGTGCTC), 2.5 µL of 10 µM reverse primer (CCGGAGCACAGACACCGCGAAG), 10 μL of 5X Q5 High GC Enhancer and 1.5 µL of Q5 DNA polymerase (New England Biolabs). The PCR was performed under the following conditions: 30 s at 98 ºC, 16 cycles of 10 s at 98 ºC, 30 s at 55 ºC and 4 min at 72 ºC and 2 min at 72 ºC.

### Cell culture

T47D, MDA-MB-468, MCF10A and HEK-293T cell lines were obtained from the ECACC commercial repository. No further authentication was conducted by the authors. Cells were negative for mycoplasma. T47D, MDA-MB-468 and HEK-293T cell lines were maintained in DMEM (AQmedia; Sigma) supplemented with 10% fetal bovine serum (FBS) (Gibco), penicillin, streptomycin and fungizone (Sigma). MCF10A was maintained in DMEM/F12 (Sigma) supplemented with 5% horse serum (Sigma), 0.02 μg/mL EGF (Sigma), 0.5 μg/mL hydrocortisone (Stem cell technologies), 10 μg/mL insulin (Sigma), 0.1 μg/mL cholera toxin (Sigma), penicillin and streptomycin.

### Transfections and plasmids

Subconfluent cells were transfected with TransIT-X2 reagent (Mirus) according to the manufacturer's instructions. At 48 h, cells were seeded in 10-cm plates with media containing the appropriate selection drug (100-450 μg/mL G418, 0.25-0.4 μg/mL puromycin). Cells were transfected with the following plasmids: pCMV6-empty vector, referred through the text as EV, pCMV6-SPN (OriGene RC213696), pBabe-puro-empty vector, pBabe-puro-p53-R175H, and pBabe-puro-YFP.

### PCR

For the confirmation of the transfection, GoTaq® Green Master Mix (Promega) was used. The PCR mixture (30 µL) contained 2 µL of the reverse transcriptase reaction product diluted 1:10, 14 µL of GoTaq® Green Master Mix, 2.5 μL of 10 µM the appropriate forward primer (c-Myc tag: GCCAGATCCTCTTCTGAGATGAG; DDK tag: CTTATCGTCGTCATCCTTGTAATC; endogenous SPN: AGGGCCGAGAAGGTAGAATC) and 2.5 µL of 10 µM reverse primer (GGCGCAGTTGGAGCAGAGTGT). The PCR was performed under the following conditions: 3 min at 95 ºC, 40 cycles of 30 s at 95 ºC, 45 s at 62 ºC and 1 min and 30 s at 72 ºC and 3 min at 72 ºC.

### RT-qPCR

Total RNA from cell lines was extracted and purified using the ReliaPrep^TM^ RNA Tissue Miniprep System (Promega), and reverse transcription was performed with 3 µg of mRNA using the High Capacity cDNA Reverse Transcription kit (Life Technologies) according to the manufacturer's instructions. The PCR mixture (10 µL) contained 2 µL of the reverse transcriptase reaction product diluted 1:10, 2.5 µL of water, 5 µL of GoTaqR Probe qPCR Master Mix (Promega) and 0.5 µL of the appropriate TaqMan Assay (20X) (Applied Biosystems). We used the following probes: *GAPDH* (Hs03929097_g1) as a endogenous control, *SPN* (Hs00261636_m1), endogenous *SPN* (AJMSHLL, customized probe using positions 2441-3000 of SPN mRNA to detect 3' UTR region), exogenous *SPN* (AJPADX1, customized probe using positions 3507-4057 of pCMV6-SPN plasmid to detect c-Myc and DDK tags), *PPP1CA* (Hs00267568_m1), *PPP1CB* (Hs01027793_m1), *PPP1CC* (Hs00160351_m1),* NANOG* (Hs04260366_g1), *SOX2* (Hs01053049_s1), *OCT4* (Hs00999632_g1), and *BMI1* (Hs00995536_m1).

### Protein isolation and western blot analysis

Western blots were performed as previously described elsewhere. Membranes were incubated with the following primary antibodies: anti-SPN (Chemicon AB5669), anti-c-Myc tag, anti-DDK tag (OriGene TA150014), anti-PP1α (Santa Cruz sc-7482), anti-PP1β (Abcam ab16369), anti-PP1γ (Abcam ab16387), anti-pRB (BD Pharmingen 554136), anti-P-pRB (Ser807/811) (Cell Signaling 9308), anti-p107 (Abnova H00005933-M01), anti-P-p107 (Ser975) (Abnova PAB4915), anti-p130 (ser672) (Abcam ab76255), anti-p53 (Santa Cruz sc-6243) and anti-α-tubulin (Sigma T9026) as a loading control. Horseradish peroxidase-labeled rabbit anti-mouse (Abcam ab97046), goat anti-rabbit (Abcam ab97051), mouse anti-rabbit IgG light chain (ab99697) and rabbit anti-sheep (Abcam ab6747) secondary antibodies were used. The proteins were detected using an ECL detection system (Amersham Biosciences) and a Bio-Rad Chemidoc Touch.

### Co-Immunoprecipitation and phosphatase assays

40 µL of protein A Sepharose (GE Healthcare) were washed twice with PBS-BSA (5 mg/mL) supplemented with a cocktail of protease and phosphatase inhibitors and then incubated with anti-SPN (1:1000), anti-pRB (BD Pharmingen 554136), anti-P-pRB (Ser807/811), anti-P-p107 (Ser975), anti-p130 (ser672) or anti-IgG (R&D Systems-105-C) antibodies in the same buffer for 3 h at 4 ºC. After 2 washes in PBS-BSA, 1 mg of cell extracts was added and incubated overnight. Immunoprecipitates were washed once with PBS-BSA and twice with IGEPAL 0.2%. Phosphatase assay was performed as previously described 29. Proteins were eluted in 40 µL of 5X Laemmli buffer (0.3 M Tris-HCl mM pH 6.8, 50% glycerol, 10% SDS, 25% 2-mercaptoethanol, 0.01% bromophenol blue), boiled 5 min and separated by 6% SDS-PAGE.

### Co-localization assays

Cells were seeded onto glass coverslips, fixed with 4% paraformaldehyde for 20 min and permeabilized with 0.5% Triton X-100 for 5 min. The coverslips were incubated with blocking solution (PBS + 0.1% Triton X-100 + 3% BSA) for 1 h and then incubated with anti-SPN antibody (1:1000) for 2 h at room temperature. The coverslips were washed four times with PBS + 0.1 Triton X-100 and incubated overnight at 4 ºC with the second primary antibody, anti-PP1α (1:200) or anti-PP1γ (1:500). Secondary antibodies anti-mouse Alexa Fluor 633 (1:500, ThermoFisher A21052), anti-rabbit Alexa Fluor 568 (1:500, ThermoFisher A11011) or anti-sheep Alexa Fluor 488 (1:1000, ThermoFisher A11015) were used. The nuclei were stained with DAPI, and the coverslips were mounted with ProLong Gold Antifade (Life Technologies). A confocal ultraspectral microscope (Leica TCS-SP2-AOBS) that allowed sequential scanning of emission channels was used for image detection.

### Growth curve

To measure the proliferation capacity, 1×10^4^ (T47D), 4×10^3^ (MCF10A) or 6×10^3^ (MDA-MB-468) cells were seeded in 12-well plates in triplicate. At 24 h (day 0), cells were fixed with 0.5% glutaraldehyde (Sigma) and every 48 h a curve point was fixed up to 15 days. Once all the points were collected, plates were stained with 1% violet crystal (Sigma). Then, the violet crystal was dissolved with 20% acetic acid (AppliChem) and the relative number of cells was quantified by measuring the absorbance of the violet crystal at 595 nm by an absorbance reader (Biorad). The values were represented referring to day 0.

### Clonogenic assay

To measure the ability of cells to form individual clones, 1×10^3^ (T47D and MCF10A) or 5x10^3^ (MDA-MB-468) cells were plated in 10 cm plates in triplicate. Cells were fixed with 0.5% glutaraldehyde and stained with 1% violet crystal after 15 days. The number of colonies was counted and types of clones classified.

### Growth in soft agar

To measure the anchorage-independent growth, cells 1x10^5^ cells were suspended in 1.4% agarose D1 Low EEO (iNtRON Biotechnology) growth medium containing 10% FBS and disposed onto 1 mL of a solidified base of 2.8% agarose growth medium in 6-well plates, in triplicate. After 24 h, 10% FBS medium was added to each well and changed twice a week. After 20-30 days, photographs were taken on an inverted microscope (Olympus IX-71) and the number of colonies was counted.

### Tumorspheres assay

1×10^3^ (T47D or MDA-MB-468) or 1×10^4^ (MCF10A) cells were seeded in triplicate in 24-well Ultra-Low Attachment Plates (Costar) containing 1 mL of MammoCult basal medium (Stem cell technologies) supplied with 10% MammoCult proliferation supplement, 4 μg/mL of heparin, 0.48 μg/mL of hydrocortisone, penicillin and streptomycin. After 5-10 days, depending on the cell line, the number of primary tumorspheres formed were measured using an inverted microscope (Olympus IX-71).

### Single-cell tumorsphere assay

Single cells were individually seeded through cell sorting with a FACS Jazz flow cytometer (BD Biosciences) in 96-well Ultra-Low Attachment Plates containing 1 mL of MammoCult basal medium (Stem cell technologies) supplied with 10% MammoCult proliferation supplement, 4 μg/mL of heparin, 0.48 μg/mL of hydrocortisone, penicillin and streptomycin. After 30 days, the number of individually primary tumorspheres formed was measured using an inverted microscope (Olympus IX-71).

### Fluorescence-activated cell sorting (FACS) analysis

For FACS analysis, 1x10^6^ cells were trypsinized and suspended in 125 µL of PBS containing 2% FBS and 5 mM EDTA. Cells were blocked with 12.5 µL of human blocking reagent (Miltenyi Biotec) for 10 min at 4 ºC. Then, cells were incubated with 5 µL of anti-CD44-FITC (Miltenyi Biotec #130-113-331) and 5 µL of anti-CD24-PE (Miltenyi Biotec #130-095-953) for 30 min at 4 ºC. After washing the cells twice with PBS-FBS-EDTA, they were suspended in 500 µL of the same buffer and analyzed by FACS with the FACS Canto II cytometer (BD Biosciences). Experiments were repeated a minimum of three times independently, in triplicate samples.

### Competition assay

Control cells were double-transfected with pCMV6-empty and pBabe-puro-empty vectors, whereas SPN-A566V cells were double-transfected with pCMV6-SPN-A566V and pBabe-puro-YFP vectors. Equal numbers (50% vs 50%) of both types of cells were seeded in the same dish, and the percentage of cells was analyzed at 24 h by FACS using the LSR II Fortessa cytometer (BD Biosciences). Then, cells were cultured for 30 days, and the final percentage of cells was counted by FACS.

### Xenograft in nude mice

Tumorigenicity was assayed by the subcutaneous injection of 8×10^6^ cells of T47D or 4×10^6^ cells of MDA-MB-468 cell lines into the right flanks of 4-week-old female athymic nude mice. Cells were suspended in 50 µL of matrigel (Corning) prior to the injection. Animals were examined weekly, after 150-180 days, depending on the cell lines, mice were sacrificed and tumors were extracted and conserved under -80 ºC. Tumorsphere tumorigenicity was measured by seeding 10^4^ cells as described in tumorspheres assay section, after 5 days tumorspheres were disaggregated with trypsin, resuspended in 50 µL of matrigel and injected into the right flanks of 4- week-old female athymic nude mice. Animals were examined weekly, after 85-100 days, depending on the cell lines, mice were sacrificed and tumors were extracted and conserved under -80 ºC. Mice inoculated with the T47D cell line were treated with 4 mg/mL of β-estradiol (Sigma) in the water bottles during all the experiments. Tumor volume (mm^3^) was measured using calipers. All animal experiments were performed according to the experimental protocol approved by the IBIS and HUVR Institutional Animal Care and Use Committee (0309-N-15).

### Cell cycle studies

Cells were synchronized in different phases of the cell cycle. For serum deprivation studies, 2×10^6^ cells were seeded in 10-cm plates, and the next day, serum was removed during 24 or 48 h. Cells were collected at different times after the addition of serum. For mimosine or nocodazole treatment, 2×10^6^ cells were seeded in 10-cm plates, and the next day, they were treated with 400 µM mimosine for 24 h or with 0.05 µg/mL nocodazole for 16-24 h. Cells were collected at different times after the treatment and analyzed by western blot or by FACS after propidium iodide staining (Sigma) and following a standard protocol.

### β-Galactosidase (X-Gal) staining

Cells were washed in PBS, fixed with 0.5% glutaraldehyde (Sigma), washed again in PBS MgCl_2_ 1 mM pH 5.5 and incubated at 37°C with fresh X-Gal staining solution containing 1.25 mg of XGal (Promega), 5 mM potassium ferricyanide (Sigma), 5 mM potassium ferrocyanide trihidrate (Sigma) in PBS MgCl_2_ 1 mM pH 5.5. Staining was evident in 4 h and then the percentage of cells expressing SA-βGal was quantified.

### Analysis of protein structure

PyMOL software (https://pymol.org/2/) was used to visualize protein structures. We selected the crystal structure of SPINOPHILIN:PP1 (PDB ID 3EGG) to simulate the mutation. FoldX software (http://foldxsuite.crg.eu/) was used to predict the effect of missense mutations in the structural stability in the structures. In this method, the stability of a protein is defined by the free energy (in kcal/mol), as calculated by the FoldX energy field in which the lower the energy, the more stable the protein. In general, if a mutation provides energy (ΔΔG > 0 kcal/mol), it will destabilize the structure. The reported accuracy of FoldX, understood as the difference between the energy calculated by FoldX and the experimental values, is 0.46 kcal/mol. Energy values can be grouped into seven categories: 1) highly stabilizing (ΔΔG <-1.84 kcal/mol), 2) stabilizing (-1.84 kcal/mol ≤ ΔΔG <-0.92 kcal/mol); 3) slightly stabilizing (-0.92 kcal/mol ≤ ΔΔG <-0.46 kcal/mol); 4) neutral (-0.46 kcal/mol <ΔΔG ≤ +0.46 kcal/mol); 5) slightly destabilizing (+0.46 kcal/mol <ΔΔG ≤ + 0.92 kcal/mol); 6) destabilizing (+ 0.92 kcal/mol <ΔΔG ≤ +1.84 kcal/mol); and 7) highly destabilizing (ΔΔG> +1.84 kcal/mol). We also checked for potential clashes, which were not apparent.

### Statistical analysis

Statistical analyses of experiments were performed using GraphPad Prism (6.01 for Windows). Control samples and SPN-A566V samples were compared using the unpaired Student's t-test or Student's t-test with Welch's correction, as appropriate. Experiments were performed a minimum of three times independently and in triplicate samples. P values less than 0.05 were considered statistically significant and were represented according to the following classification: p <0.05 (*), p <0.01 (**), and p <0.001 (***).

## Results

### Mutational analysis: search for mutations in SPN in human tumors

**Figure 1:**
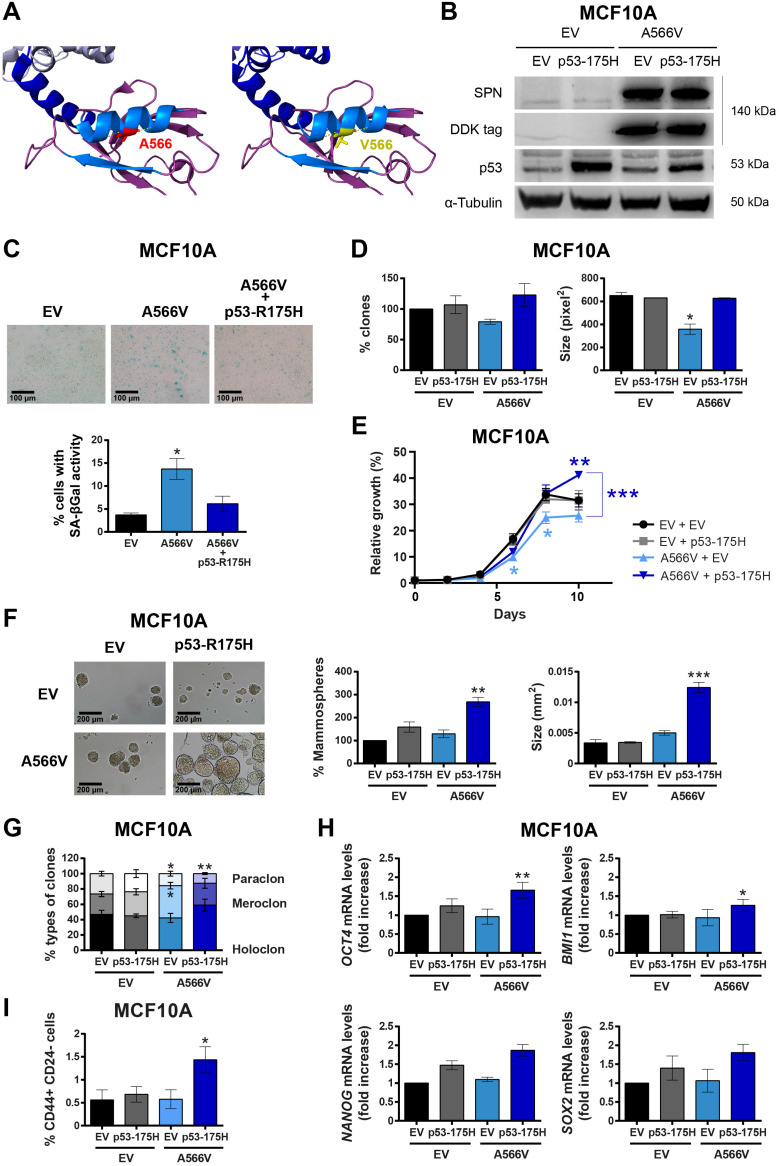
** The SPN-A566V mutation induces stemness in the MCF10A cell line when p53 is mutated. A)** Analysis of the structural impact of the SPN-A566V mutation using FoldX and PyMOL software. Structure of SPINOPHILIN:PP1 (PDB ID 3EGG) is represented: PP1 protein (gray), the PDZ domain of SPN (purple) and the PP1-binding domain of SPN (blue). The second alpha helix and the second beta sheet of the PDZ domain are highlighted in light blue. The original residue A566 is in red, and the mutated V566 is in yellow. **B)** Validation of the double transfection of pCMV6-EV or pCMV6-SPN-A566V with pBabe-EV and pBabe-p53-R175H in the MCF10A cell line by western blot analysis using the DDK-tag, anti-SPN and anti-p53 antibodies. **C)** Upper, representative images of SA-β-gal activity are shown (scale bars: 100 µm). Bottom, percentage of cells with SA-β-gal activity in MCF10A cells with empty vector (EV), SPN-A566V mutant (A566V) or with both SPN-A566V and p53-R175H mutants. **D)** Clonogenic assay of MCF10A control and SPN-A566V cell lines in the context of mutated p53. Cells were seeded at low density, and after 10 days, the number of colonies was counted, and the size was measured. **E)** Growth curves of MCF10A control and SPN-A566V cell lines in the context of mutated p53. **F)** Percentage and size of mammospheres formed by MCF10A control and SPN-A566V cell lines in the context of mutated p53. Representative images of the mammospheres are shown (scale bars: 200 µm). **G)** Percentages of holoclones, meroclones and paraclones generated by MCF10A control and SPN-A566V cell lines in the context of mutated p53 seeded at low density for 10 days. **H)** Measurement of *NANOG, OCT4, BMI1* and *SOX2* expression levels by RT-qPCR in MCF10A control and SPN-A566V cell lines in the context of mutated p53. Graphs represent mRNA levels normalized to the mRNA levels of control cells (EV + EV). **I)** Quantification of the percentages of CD44+ CD24- cells in MCF10A control and SPN-A566V cell lines in the context of mutated p53 by FACS. The mean of a minimum of 3 independent experiments performed in triplicate ± standard deviation is represented. Statistical analysis was performed with the t-Student test**,** * p < 0.05, ** p < 0.01, *** p < 0.001.

We found 122 mutations in the SPN protein described in human tumors throughout the protein sequence, with those that could affect the interaction of SPN with PP1 and pRB being of special interest **(Figure S1)**. We performed our own mutational analysis using 30 different samples from cancer cell lines and tumor samples focusing only on the region of interaction with PP1. We found a silent mutation, P456, and a missense mutation, A566V **(Figure 1A and Figure S2)**. Interestingly, the SPN-A566V mutation was also reported in the cBioPortal database, along with 38 other mutations in the SPN/PP1 interaction region **(Table S1 and Figure S1)**. In this mutational analysis, we found that around 50% of tumors that carry a mutation in SPN, also present an inactivating mutation in p53 **(Table S2)**. Additionally, the majority of those tumors with wild-type p53 also carry mutations capable to inactive the p53 pathway. SPN-A566V is located in the PDZ domain of SPN, specifically in an alpha helix through which multiple proteins interact 26. Then, we performed an in-depth analysis to estimate the structural impact of the mutation selected using FoldX software. According to our predictions, the SPN-A566V mutation would have no effect on protein stability, nor would it produce losses or gains of interactions with PP1. However, as the residue A566 is located within the interface of an interaction surface, the mutation to a bulkier valine could affect the interaction of SPN with other proteins **(Figure 1A)**. Therefore, A566V mutation would not reduce the levels of the protein but may alter its activity, probably through PP1 binding.

### Effect of SPN-A566V in an immortalized non-tumorigenic breast cell line

To study the effect of SPN-A566V in vitro, we overexpressed mutated SPN-A566V or an empty vector (pCMV6-EV) as a control in the immortalized non-tumorigenic cell line of epithelial breast tissue, MCF10A 30. This overexpression maintaining wild-type alleles would be, to certain extent, equivalent to tumoral heterozygous mutation of SPN. The overexpression of SPN-A566V was validated at protein level by western blot using the DDK-tag. The overexpressed mutated protein was maintained and stabilized and not degraded **(Figure 1B)**. Since the MCF10A cell line expresses wild-type p53 and the loss of SPN has been associated with p53 mutations 2,3,12,17, we also overexpressed a mutated p53 protein, p53-R175H, and an empty vector as a control (pBabe-EV) in both control and SPN-A566V-overexpressing cells **(Figure 1B)**. First, we analyzed the percentage of cells that enter in senescence by measuring the senescence associated β-Galactosidase activity (SA-βGal). We found that the overexpression of SPN-A566V mutation induces an increase in the percentage of cells with SA-βGal activity, whereas the overexpression of SPN-A566V and p53-R175H mutations at the same time did not induce this senescent phenotype **(Figure 1C)**. We performed a clonogenic assay to analyze the ability of cells to form colonies in the absence of cellular contact, and we observed that cells with the SPN-A566V mutation formed fewer and smaller colonies than control cells. However, cells that overexpress both mutations, SPN-A566V and p53-R175H, formed higher numbers and larger colonies than cells with only the SPN-A566V mutation **(Figure 1D)**. MCF10A cells with SPN-A566V also grew slowly than control cells. Instead, cells that overexpressed the SPN-A566V mutation grew faster when they had mutated p53, eventually growing more than control cells **(Figure 1E)**.

Then, we explored the effects of both mutations on the stemness capability of the MCF10A cell line. We performed a serum-free 3D suspension “mammosphere” culture assay in the MCF10A cell line. We found that those cells with SPN-A566V and p53-R175H mutations were able to form a higher number of and larger mammospheres than control cells or those with SPN-A566V and wild-type p53 **(Figure 1F)** and, therefore, they were enriched in epithelial progenitors with higher expression of CSC markers. By measuring the phenotypes of the clones formed after seeding the cells at low density 31-33, we observed an increase in the percentage of holoclones (colonies enriched in CSCs) and a decrease in the percentage of paraclones (colonies enriched in mature, non-stem cells) in cells that overexpress SPN-A566V and mutated p53 **(Figure 1G)**. Furthermore, these cells expressed higher mRNA levels of some CSC markers such as NANOG, SOX2, OCT4 and BMI1 than control or SPN-A566V wild-type p53 cells **(Figure 1H)** and showed a higher proportion of CD44+ CD24- cells, which are cancer-initiating cells in breast tumors **(Figure 1I)** 20,21,34.

Our results confirm that p53 inhibition is necessary to bypass certain culture arrest induced by the SPN-A566V mutant. These data are in accordance with previous studies in vivo using Spn knock-out mice, in which the loss of Spn needs the mutation of p53 to induce full tumorigenesis in the mammary glands of mice 2,3.

### Effect of SPN-A566V in the tumorigenic properties of breast cancer cells

**Figure 2:**
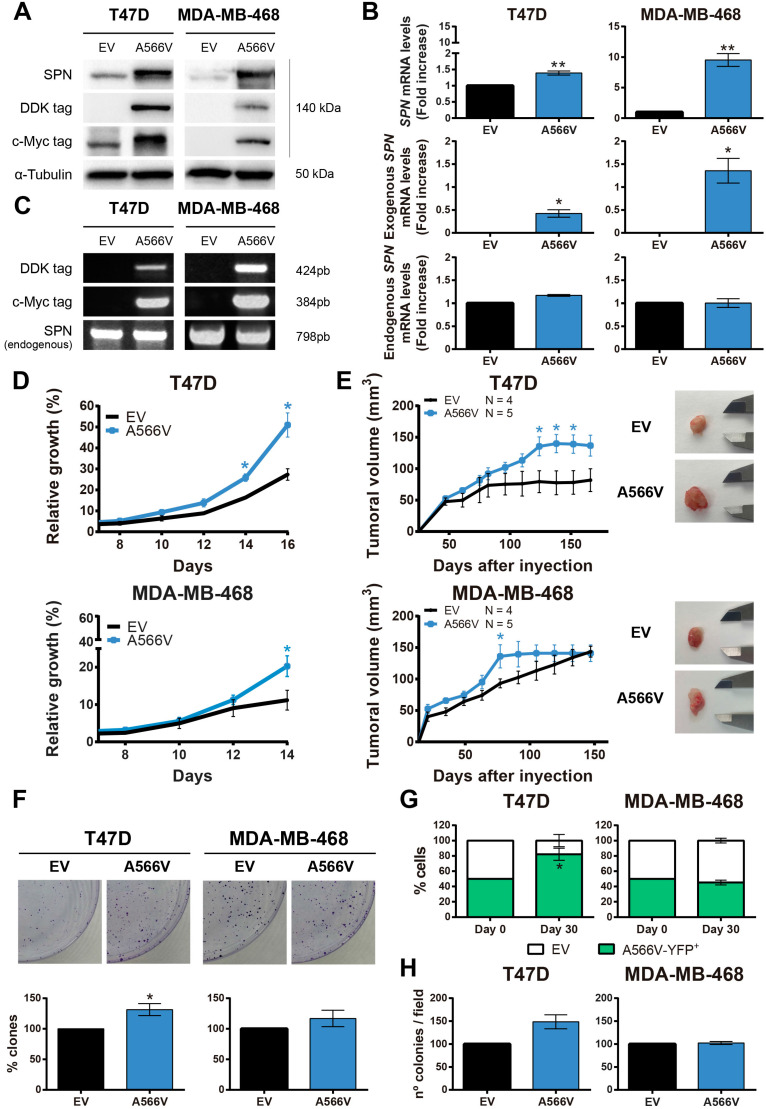
** The SPN-A566V mutation increases tumorigenesis in breast cancer cell lines *in vitro* and *in vivo*. A-C)** Validation of the overexpression of SPN-A566V in the T47D and MDA-MB-468 breast cancer cell lines by western blot analysis **(A)**, RT-PCR **(B)** or PCR** (C)** measuring endogenous SPN and exogenous SPN using different tags. Cells were transfected with an empty vector (EV) as a control or with the mutation of SPN (SPN-A566V). **D)** Growth curves of T47D and MDA-MB-468 control and SPN-A566V cell lines. **E)** Tumor growth in xenografts from T47D and MDA-MB-468 control and SPN-A566V cell lines. Cells were injected in nude mice (EV N=4, A566V N=5), and tumor size was measured weekly. Mice inoculated with the T47D cell line were treated with 4 mg/mL of β-estradiol. Graphs represent the tumor size (mean ± SEM). Representative images of tumor size are shown. **F)** Clonogenic assay of T47D and MDA-MB-468 control and SPN-A566V cell lines. Cells were seeded at low density, and after 15 days, colonies were counted. Representative images are shown.** G)** Competition assay of T47D and MDA-MB-468 control and SPN-A566V-YFP+ cell lines. Equal numbers of both types of cells were seeded, and after 30 days, the percentage of YFP+ cells was measured by FACS. **H)** Soft agar assay of T47D and MDA-MB-468 control and SPN-A566V cell lines. After 30 days, colonies were counted. The mean of a minimum of 3 independent experiments performed in triplicate ± standard deviation is represented in all experiments. Statistical analysis was performed with the t-Student test, * p < 0.05, ** p < 0.01.

SPN-A566V seems to potentiate the tumorigenic and stemness properties of the cells depending on the molecular state of p53. To study the specific p53-independent effect of this mutation in breast cancer, we focused on two different breast cancer cells lines that carry p53 mutations, T47D and MDA-MB-468 **(Figure S3)**. We used these two cells lines with different molecular expression of pRB since the role of SPN as a tumor suppression gene seems to depend on PP1 and pRB to analyze if other members of the pocket protein family could compensate pRB functions. We overexpressed the SPN-A566V mutation and an empty vector as a control in these two cell lines. The overexpression was validated at the protein level by western blot analysis **(Figure 2A)** and at the mRNA level by RT-qPCR **(Figure 2B)** and by PCR **(Figure 2C)** using the tags of the exogenous overexpressed SPN. First, we found that cells overexpressing SPN-A566V grew faster than control cells in both cell lines **(Figure 2D)**. Indeed, when cells were injected as xenografts in nude mice, cells with the mutation of SPN formed larger tumors and they grew more rapidly than the ones formed by control cells **(Figure 2E)**. We also observed that SPN-A566V cells formed a higher number of colonies than control cells **(Figure 2F)**. Additionally, we evaluated how different cell populations compete under the same conditions. For this, we double-transfected cells with two empty vectors and SPN-A566V cells with pBabe-puro-YFP (yellow fluorescent protein) and selected for double expression. Equal numbers of both cells were seeded in the same dish and, after 30 days, we observed that T47D-A566V cells increased their pool, with a stable higher percentage of the mixed population **(Figure 2G)**, indicating a competitive advantage over cells without the mutation. We also performed a soft agar assay to measure anchorage-independent cell growth and found that T47D-A566V cells formed a higher number of colonies than control cells **(Figure 2H)**. Therefore, we conclude that the SPN-A556V mutation increases the tumorigenic properties of p53-mutated breast cancer cells both in vitro and in vivo.

We also studied the cell cycle in T47D and MDA-MB-468 control and SPN-A566V cell lines. We synchronized cells at G0 by serum deprivation for 24 h and we measured the percentage of cells in each phase of the cell cycle at different time points by FACS **(Figure S4)**. We observed that T47D-SPN-A566V cells showed a higher percentage of cells in S phase at 16 h than control cells **(Figure S4A)**. Additionally, faster growing MDA-MB-468-SPN-A566V cells had a greater number of cells in G2 phase at 8 h than control cells **(Figure S4C)**. We did not observe sub-G0 cells in any cell line, indicating that apoptosis was not taking place **(Figure S4B and S4D)**. Therefore, these results suggest that cells that overexpress the SPN-A566V mutation are entering in S and G2 phases earlier and cycling faster than control cells.

### Stemness capability of SPN-A566V cells

**Figure 3:**
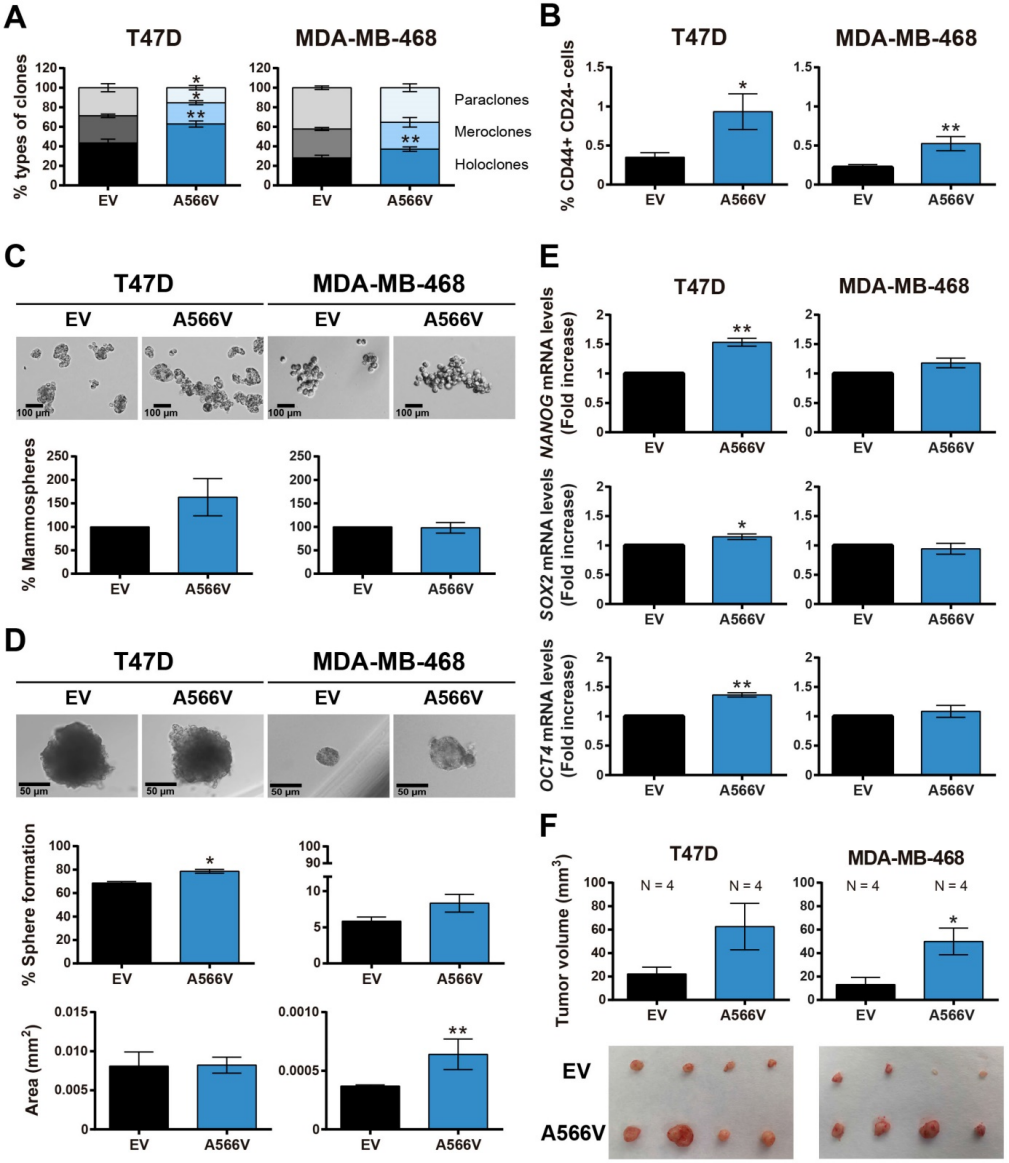
** The SPN-A566V mutation induces stemness in breast cancer cell lines. A)** Percentages of holoclones, meroclones and paraclones generated by T47D and MDA-MB-468 control and SPN-A566V cell lines seeded at low density over 15 days. **B)** Quantification of the percentages of CD44+ CD24- cells in T47D and MDA-MB-468 control and SPN-A566V cell lines by FACS. **C)** Percentages of mammospheres formed from the whole population of T47D and MDA-MB-468 control and SPN-A566V cell lines. Representative images of the mammospheres are shown (scale bars: 100 µm). **D)** Percentages of mammospheres formed from one single cell of T47D and MDA-MB-468 control and SPN-A566V cell lines separated by FACS. Graphs represent the quantification of the number and size of mammospheres. Representative images of the mammospheres are shown (scale bars: 50 µm). **(E)** Measurement of *NANOG, SOX2* and *OCT4* expression levels by RT-qPCR in T47D and MDA-MB-468 control and SPN-A566V cell lines. Graphs represent mRNA levels in SPN-A566V cells normalized to the mRNA levels of control cells. The mean of a minimum of 3 independent experiments performed in triplicate ± standard deviation is represented in all experiments. **(F)** Tumorigenicity of tumorspheres *in vivo*. Mammospheres from T47D and MDA-MB-468 control and SPN-A566V cells were injected in nude mice (N = 4). Mice inoculated with the T47D cell line were treated with 4 mg/mL of β-estradiol. Graphs represent the tumor size (mean ± SEM). Representative images of tumor size are shown. Statistical analysis was performed with the t-Student test, * p < 0.05, ** p < 0.01.

To explore the effect of the SPN-A566V mutation on the stemness capability of the cells, we performed a clonability assay to measure the phenotypes of the clones and observed that cells that overexpress the SPN mutation formed a higher number of holoclones and a lower number of paraclones than control cells **(Figure 3A)**. In addition, cells with the SPN-A566V mutation showed a higher proportion of CD44+ CD24- cells **(Figure 3B)**. We also observed that cells with the SPN-A566V mutation formed a higher number of mammospheres both when they were seeded from the whole population **(Figure 3C)** or from single-cell sorting **(Figure 3D)**. The single-cell mammospheres formed by MDA-MB-468-SPN-A566V cells were larger than control cells **(Figure 3D)**. Indeed, those cells with the mutation of SPN expressed higher mRNA levels of some CSC markers, such as NANOG, SOX2 and OCT4 **(Figure 3E)**. Finally, we injected the mammospheres from SPN-A566V and control cells into nude mice. After 85-100 days, depending on the cell line, we observed that mammospheres from both cell lines formed tumors and that those tumors formed from SPN-A566V cells were larger than those formed from control cells **(Figure 3F)**. These results indicate that p53-mutated breast cancer cells show increased stemness when carrying the SPN-A566V mutation.

### Effect of SPN-A566V in the interaction with PP1

**Figure 4:**
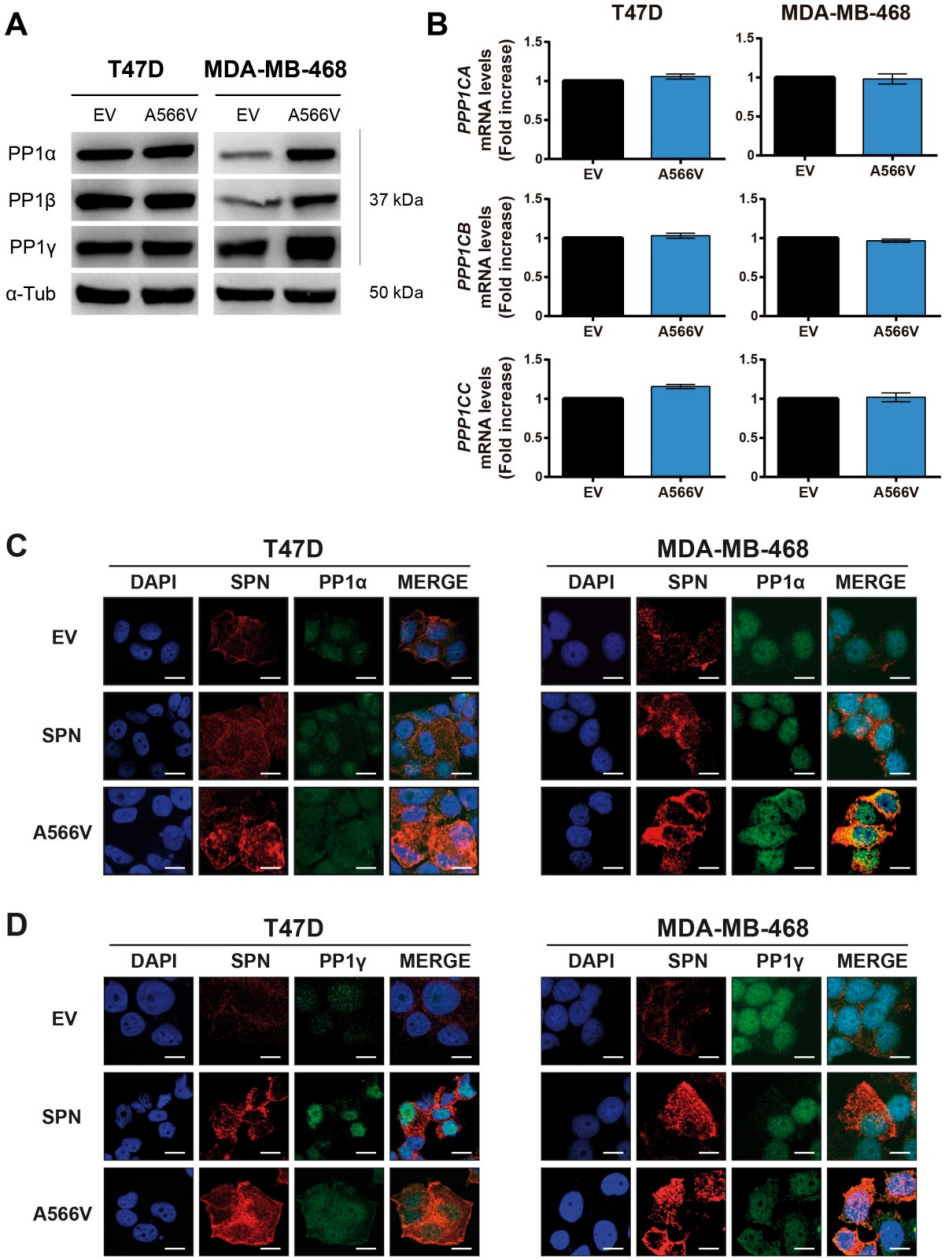
** Effect of SPN-A566V in the levels and localization of PP1. A-B)** Measurement of the levels of the three catalytic subunits of PP1 by western blot analysis **(A)** or by RT-qPCR** (B)** in control and SPN-A566V cells. The mean of a minimum of 3 independent experiments performed in triplicate ± standard deviation is represented. Statistical analysis was performed with the t-Student test.** C-D)** Co-localization assay of SPN and PP1α **(C)** or PP1γ **(D)** in control cells (EV), cells that overexpress wild-type SPN or cells with the mutation SPN-A566V. Cells were stained using SPN, PP1α and PP1β antibodies and DAPI as a nuclear control (scale bars: 10 µm).

**Figure 5:**
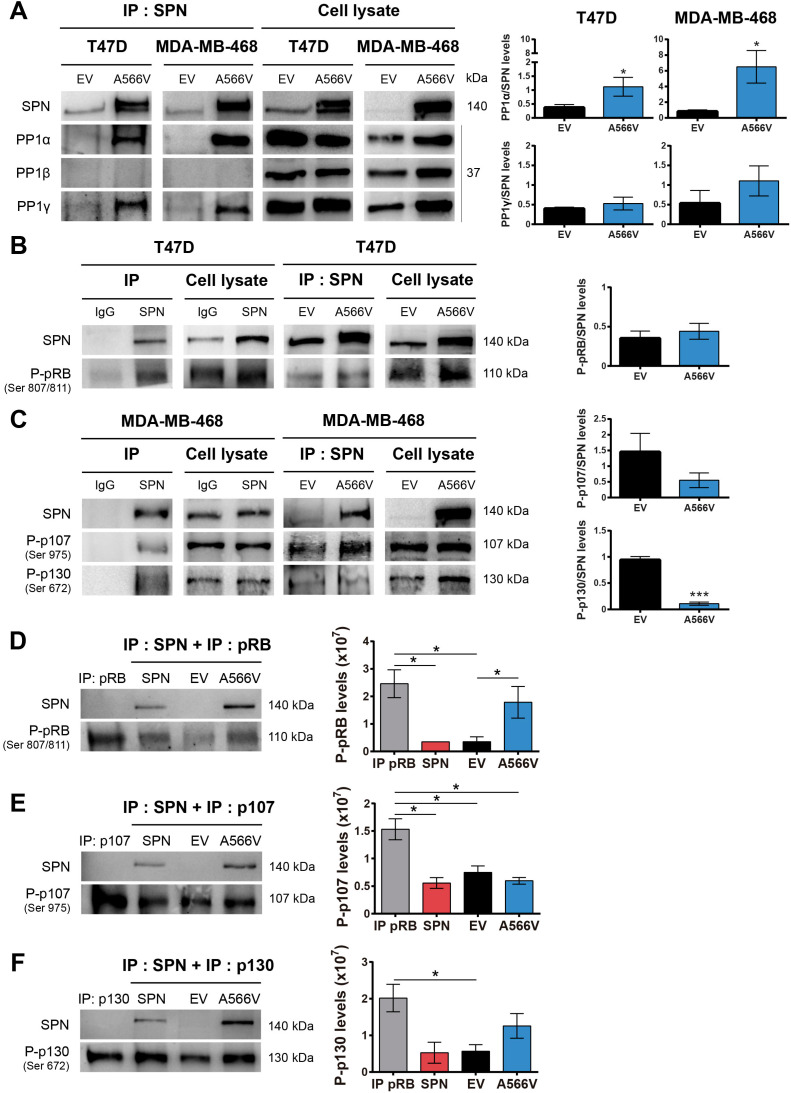
** The interaction between SPN and PP1 and the phosphatase activity of the holoenzyme are compromised by the SPN-A566V mutation. A)** Co-immunoprecipitation of SPN and the three catalytic subunits of PP1 in control and SPN-A566V cells. Protein extracts from T47D and MDA-MB-468 cells were subjected to immunoprecipitation with anti-SPN, and the immunoprecipitates were analyzed with anti-SPN, anti-PP1α, anti-PP1-β and anti-PP1γ antibodies. **B-C)** Co-immunoprecipitation of SPN and pocket proteins in control and SPN-A566V cells. Protein extracts from T47D and MDA-MB-468 cells were subjected to immunoprecipitation with anti-SPN or anti-IgG antibodies, and the immunoprecipitates were analyzed with anti-P-pRB ser807/811 **(B)**, anti-P-p107 ser975 and anti-P-p130 Ser 672 **(C)** antibodies. Proteins that are not immunoprecipitated were included as a control (cell lysate). **D-F)** Phosphatase assay of the PP1-SPN holoenzyme. HEK293T cells were transiently transfected with wild-type SPN (lane 2), SPN-A566V (lane 4) and the empty vector (lane 3), and then SPN was immunoprecipitated. At the same time, P-pRB **(D)**, P-p107 **(E)** and P-p130 (lane 1) **(F)** were immunoprecipitated in the nontransfected parental HEK-293T cells being used as substrates over 40 min. The results were analyzed by western blot analysis (left panel) and quantified (right panel). A representative image of 3 experiments performed independently is shown. The mean of a minimum of 3 independent experiments performed in triplicate ± standard deviation is represented in all experiments. Statistical analysis was performed with the t-Student test, * p < 0.05.

Since SPN is a PP1 regulatory protein, we decided to study how this mutation could affect the interaction with PP1 in vitro. First, we observed that SPN-A566V did not significantly change the expression levels of the three catalytic subunits of PP1, either at the protein **(Figure 4A)** or mRNA levels **(Figure 4B)**. Next, we analyzed the co-localization of SPN-A566V and PP1. As it has been described that SPN does not interact with PP1β 35, we focused on the co-localization with PP1α and PP1γ. We observed that SPN was preferentially located in the cytoplasm and that PP1α and PP1γ were located in the nucleus in control cells and cells that overexpress wild-type SPN, whereas there was a co-localization of SPN and PP1α or PP1γ in the nucleus and in the cytoplasm of those cells that overexpress the SPN-A566V mutant **(Figure 4C and 4D)**. This result suggests that the mutation SPN-A566V induces a partial change in the subcellular localization of PP1. Then, we performed a co-immunoprecipitation assay to analyze if the mutation of SPN affects the interaction between SPN and PP1. We found that SPN-A566V interacts strongly with PP1α and PP1γ. In addition, like wild-type SPN, SPN-A566V does not interact with PP1β **(Figure 5A)**.

One of the main substrates of PP1 is the retinoblastoma protein (pRB), which controls the expression of genes involved in the cell cycle 36,37. PP1 forms a complex preferentially with pRB, but it can also binds P-pRB through a PP1 regulatory protein 4,38-42. Previous studies demonstrated that SPN is involved in the PP1-dependent dephosphorylation of pRB 2,5. For that reason, we analyzed the interaction between SPN-A566V and P-pRB by co-immunoprecipitation, focusing on serine residues 807/811, two of the preferred dephosphorylation sites of PP1 43. We only studied this interaction in T47D cells because MDA-MB-468 cells do not express pRB **(Figure S3)**. We found that SPN interacts with P-pRB in a specific manner; however, this interaction is not compromised by the mutation of SPN **(Figure 5B)**. Next, we decided to study the interaction of SPN with the other two pocket proteins, p107 and p130, in the MDA-MB-468 cell line to determine if other members of this family can interact with SPN and PP1. To date, the interaction of PP1 with p107 or p130 and the ability of PP1 to dephosphorylate them have not been described. Therefore, we analyzed the interaction between SPN-A566V and P-p107 by co-immunoprecipitation, focusing on serine 975, a homologous residue to serines 807/811 of P-pRB. We also analyzed the interaction between SPN-A566V and P-p130, focusing on serine 672 since it is an important residue implicated in the stability of p130 during the cell cycle and a possible dephosphorylation site by PP1 44-47. We found that SPN interacts with both P-p107 and P-p130 in a specific manner and that SPN-A566V interacts with both of them **(Figure 5C)**.

To explore the effect of the mutation on the activity of the holoenzyme PP1-SPN, we performed an in vitro phosphatase assay. We used the HEK-293T cell line that expresses low levels of SPN. After a transient transfection with wild-type SPN, SPN-A566V or the empty vector, SPN was immunoprecipitated. We also immunoprecipitated either P-pRB, P-p107 or P-p130 in the nontransfected parental HEK-293T cells, and we used these immunoprecipitates as substrates for the corresponding dephosphorylation reactions of each SPN immunoprecipitated in complex with PP1. In the P-pRB phosphatase assay, we observed that P-pRB is dephosphorylated in serine 807/811 by the holoenzyme formed by PP1-endogenous SPN (control cells) and by PP1-wild-type SPN. However, the holoenzyme containing the mutation, PP1-SPN-A566V, showed ability to bind both total pRB **(Figure S5)** and P-pRB and reduced ability to dephosphorylate the later in serine 807/811 **(Figure 5D)**. In the P-p107 phosphatase assay, we observed for the first time that PP1 in complex with wild-type SPN is able to partially dephosphorylate P-p107 in serine 975. However, the ability of this holoenzyme to dephosphorylate P-p107 is not compromised due to the SPN-A566V mutation **(Figure 5E)**. Finally, in the P-p130 phosphatase assay, we also observed for the first time that PP1 in complex with SPN is able to partially dephosphorylate P-p130 in serine 672. Indeed, the holoenzyme PP1-SPN-A566V showed lower ability to dephosphorylate P-p130 than the holoenzyme formed by PP1 and wild-type SPN **(Figure 5F)**. Therefore, our results show that the SPN-A566V mutation modifies the interaction between SPN and PP1, and this affects the phosphatase activity of the holoenzyme, especially in the dephosphorylation of the pocket proteins P-pRB and P-p130.

### Effect of SPN-A566V in the ability to dephosphorylate pocket proteins during the cell cycle

SPN is thought to play an important role in the dephosphorylation of pRB during the cell cycle through regulation of PP1 2,5. Since cells that overexpress SPN-A566V have increased tumorigenic and stemness properties, we analyzed the effect of this mutation in the ability of the holoenzyme to dephosphorylate pocket proteins during the cell cycle.

**Figure 6:**
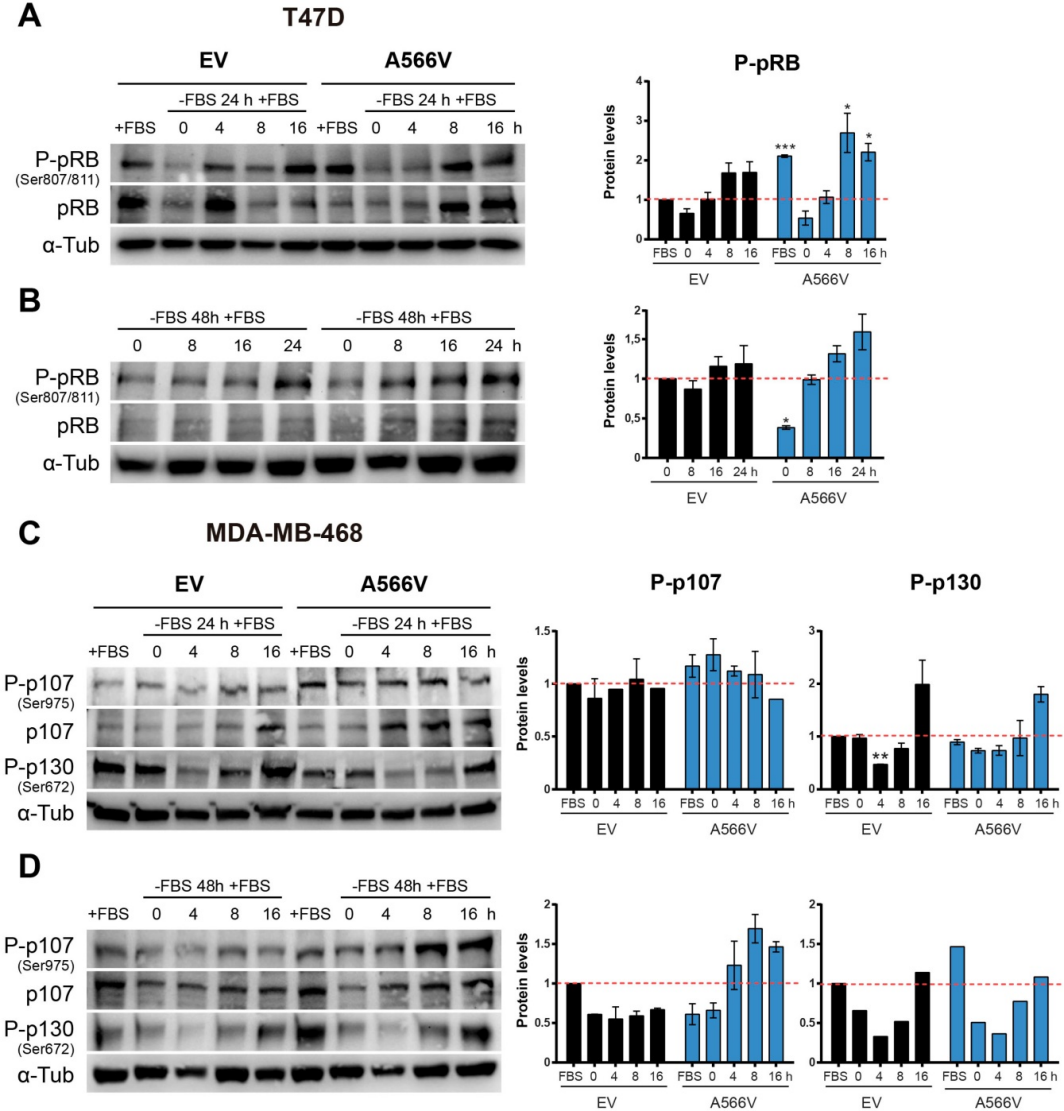
** The SPN-A566V mutation induces an early recovery from serum deprivation through the deficient dephosphorylation of pocket proteins. A-B)** Measurement of the levels of total and phosphorylated (Ser 807/811) pRB in T47D control and SPN-A566V cells after serum deprivation for 24 **(A)** or 48 h **(B)** by western blot analysis. **C-D)** Measurement of the levels of total and phosphorylated (Ser975) p107 and phosphorylated (Ser672) p130 in MDA-MB-468 control and SPN-A566V cells after serum deprivation for 24 **(C)** or 48 h **(D)** by western blot analysis. Cells were seeded, and 24 h later, serum was eliminated for 24 or 48 h, and cells were collected at different points after serum addition. *Left,* representative images of western blot analysis are shown; *right,* protein levels were quantified and normalized according to α-tubulin levels and to the first point. The mean of a minimum of 3 independent experiments performed in triplicate ± standard deviation is represented. Statistical analysis was performed with the t-Student test, * p < 0.05, ** p < 0.01, *** p < 0.001.

First, we synchronized cells at G0 by serum deprivation during 24 or 48 h, and we measured the ability of cells to recover by adding new growth factors. Levels of total and phosphorylated pRB were measured at different time points **(Figure 6A-B)**. We observed that T47D cells overexpressing SPN-A566V showed higher levels of P-pRB than control cells when they grew without serum restrictions **(Figure 6A)**. In addition, T47D cells with SPN mutation showed a stronger recovery from 24-h serum withdrawal, as they had higher levels of both total and P-pRB at 8 and 16 h after the addition of serum **(Figure 6A)**. In the case of 48-h serum deprivation, we also observed that T47D cells that overexpressed SPN-A566V had an earlier recovery than control cells **(Figure 6B)**. As MDA-MB-468 cells do not express pRB, we measured the levels of the other two pocket proteins, p107 and p130, in this cell line in the same conditions. We found that MDA-MB-468 cells that overexpress SPN-A566V had higher levels of P-p107 when cells grew without serum restrictions. These cells also showed higher levels of P-p107 at 0, 4 and 8 h and higher levels of total p107 at 4, 8 and 16 h after 24-h serum deprivation. However, we did not observe differences in the phosphorylation of p130 **(Figure 6C)**. In the case of 48-h serum deprivation, we also observed that MDA-MB-468 cells that overexpressed SPN-A566V had an earlier recovery than control cells. Furthermore, in this case, we also observed an increased phosphorylation of p130 in cells that overexpress SPN-A566V when they grew without serum restrictions **(Figure 6D)**. These data suggest that SPN-A566V is implicated in the dephosphorylation of P-pRB and P-p107, and partially P-p130, during G0/G1 transition.

**Figure 7:**
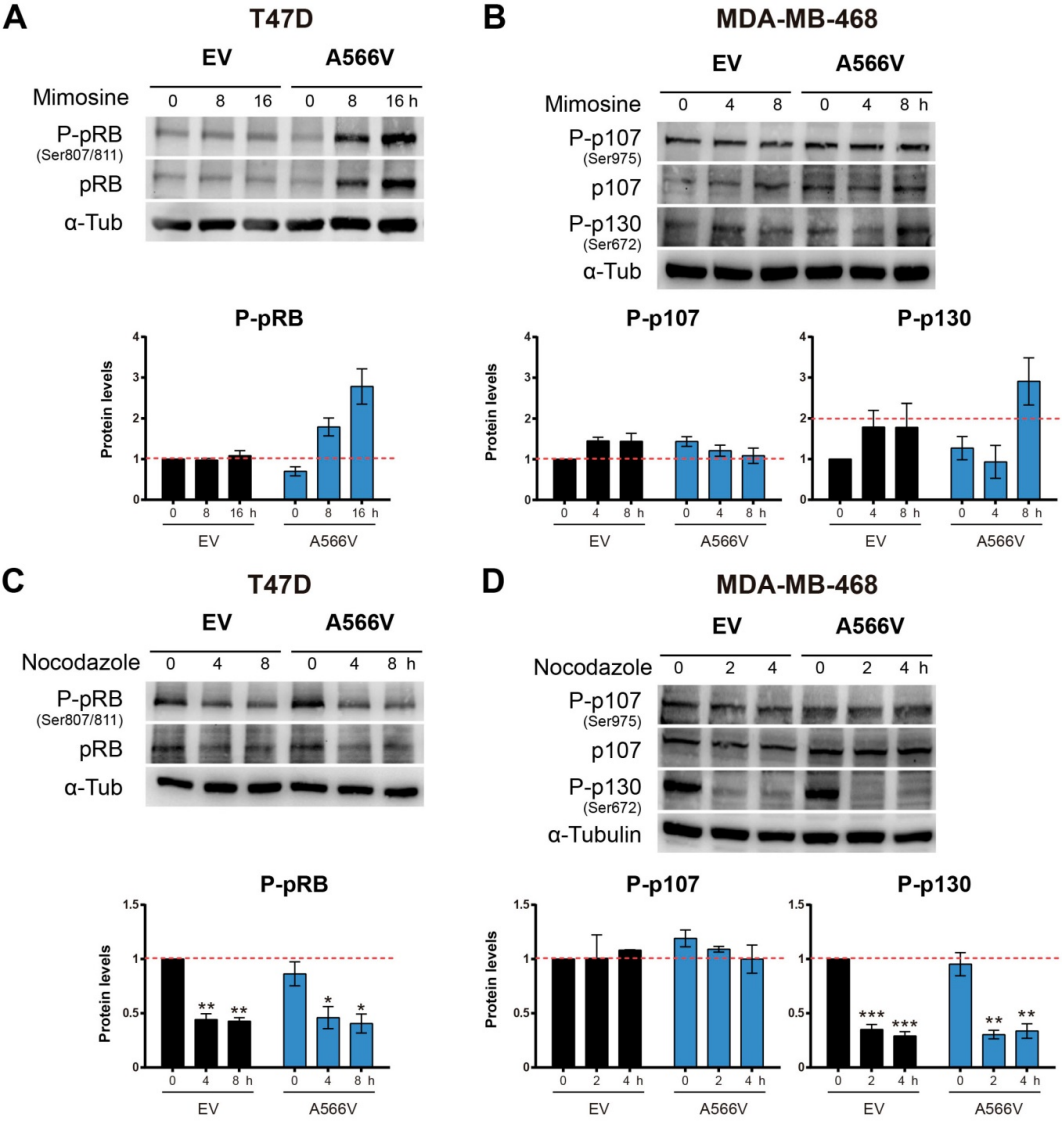
** The SPN-A566V mutation regulates the dephosphorylation of pocket proteins during the end of G1 but not during G2/M A)** Measurement of the levels of total and phosphorylated (Ser 807/811) pRB in T47D control and SPN-A566V cells after mimosine treatment for 24 h. **B)** Measurement of the levels of total and phosphorylated (Ser975) p107 and phosphorylated (Ser672) p130 in MDA-MB-468 control and SPN-A566V cells after mimosine treatment for 24 h. **C)** Measurement of the levels of total and phosphorylated (Ser 807/811) pRB in T47D control and SPN-A566V cells after nocodazole treatment for 24 h.** D)** Measurement of the levels of total and phosphorylated (Ser975) p107 and phosphorylated (Ser672) p130 in MDA-MB-468 control and SPN-A566V cells after nocodazole treatment for 16 h. Cells were seeded, and 24 h later, treatment was applied; cells were collected at different points after treatment ended. *Upper,* representative images of western blot analysis are shown; *bottom,* protein levels were quantified and normalized according to α-tubulin levels and to the first point. The mean of a minimum of 3 independent experiments performed in triplicate ± standard deviation is represented in all experiments. Statistical analysis was performed with the t-Student test, * p < 0.05, ** p < 0.01, *** p < 0.001.

Next, we synchronized cells at late G1 through mimosine treatment. Cells were seeded and treated after 24 h with 400 µM mimosine during 24 h. Then, levels of the pocket proteins were measured at different time points **(Figure 7A-B)**. We observed that T47D cells overexpressing SPN-A566V showed higher levels of total and P-pRB at 8 and 16 h after mimosine treatment **(Figure 7A)**. Conversely, we found that in the MDA-MB-468 cell line, the levels of total and P-p107 remained constant over time in both control and SPN-A566V cells. However, MDA-MB-468 cells overexpressing SPN-A566V showed higher levels of P-p130 at 8 h after treatment ended **(Figure 7B)**. These data suggest that SPN-A566V plays an important role in the dephosphorylation of P-pRB and, partially, in the dephosphorylation of P-p130 at the end of G1.

Then, we synchronized cells at the G2/M transition through nocodazole treatment. Cells were seeded, and 24 h later, they were treated with 0.05 µg/mL nocodazole for 16 (MDA-MB-468) or 24 h (T47D). Levels of the pocket proteins were measured at different time points **(Figure 7C-D)**. We did not observe changes in the phosphorylation levels of pocket proteins between control and SPN-A566V cells in either the T47D **(Figure 7C)** or MDA-MB-468 **(Figure 7D)** cell lines. These data suggest that SPN is not implicated in the dephosphorylation of pocket proteins during the end of G2 and mitosis. Therefore, we conclude that the function of SPN in the dephosphorylation of pocket proteins occurs exclusively during G0/G1 and G1/S transitions.

## Discussion

SPN, a gene located at locus 17q21.33, is an important tumor suppressor involved in the progression and malignancy of many tumors, including breast cancer 5-11,48. In vivo studies demonstrated that Spn-/- mice are more likely to develop breast tumors 3. Furthermore, SPN levels are reduced or lost in 15% of breast tumors, correlating with a higher histological grade and p53 mutations. The loss of SPN increases the CSC phenotype in breast tumors through interaction with PP1 and pRB 17. We have characterized an oncogenic mutation of SPN found in human tumors, A566V, which affects both the interaction SPN-PP1 and the phosphatase activity of the holoenzyme. Our data reveal that SPN-A566V would be an event that promotes context-dependent tumorigenesis by inducing the CSC pool in breast tumors.

Human tumors show mutations in SPN at a low frequency. The A566 residue is located in the PDZ domain of SPN, specifically in an alpha helix that is part of the interaction surface through which SPN interacts with multiple proteins 49,50. The estimation data of the structural impact of the mutation suggest that SPN-A566V would have no effect on protein stability nor produce losses or gains of interactions with PP1. Therefore, although SPN-A566V would not potentially affect PP1 binding, it could compromise the binding of other proteins to the PDZ domain, such as pRB, since the added valine is a bulkier residue that would reduce the useful space for interaction (Figure 1A) 26. The frequency found for such SPN mutations in human tumors is very low but may be in accordance with the high ratio of other mutations found in the pRB pathway (i.e., pRB, INK4a loss, or SMAD), or loss of heterozygosity of the SPN loci 17.

In vivo studies suggest a relationship between SPN and p53, similarly to pRB and p53, since the loss of both genes increased the incidence of tumors (e.g., lymphomas) and neoplastic lesions in the mammary glands 3. In fact, Spn-/- mice expressing p53-R172H exhibited significant increases in branching and alveolar growth and a higher percentage of breast tumors 3. The loss of SPN in human tumors has also been associated with p53 mutations 2,3,12,17. This loss induces a proliferative response by reducing the levels of PP1 and increasing the levels of inactive P-pRB 2,5. At the same time, a neutralization of this proliferative response through p53/ARF activities is produced. In this way, once p53 is mutated, the proliferative response and the tumorigenic properties of cells are potentiated 2,5,17. In our mutational analysis, we found that around 50% of tumors that carry a mutation in SPN, also present an inactivating mutation in the p53 gene, and the majority of those tumors with wild-type p53 also carry mutations capable to inactivate p53. Indeed, the inactivation of the p53 pathway can occur by several mechanisms equally altered in human tumors. Some of these mechanisms are MDM2 amplification, deletion or methylation of CDKN2A (p14ARF), unbalanced NOTCH pathway, non-coding specific microARNs, etc 51. Therefore, although mutations in p53 is the main proof of concept of the necessity to inactivate p53 in the case of loss or mutation of SPN, in fact this inactivation may occur by diverse mechanisms equally active and reported in human tumors (Table S2). We confirmed this dependence of inactive p53 for the effect of the SPN-A566V mutation in tumorigenesis by using an immortalized non-tumorigenic cell line of epithelial breast tissue that expresses wild-type p53, MCF10A 30. We found that the overexpression of SPN-A566V mutation induces an increase in the percentage of cells with SA-βGal activity, whereas the overexpression of SPN-A566V and p53-R175H mutations at the same time did not induce this senescent phenotype. Indeed, cells with both SPN-A566V and p53-R175H mutations formed more and larger colonies and grew faster than cells with only SPN-A566V, probably as a result of senescence bypass. In addition, an increase in the stemness properties (number of holoclones, mammospheres, CD44+ CD24- cells and expression of some CSC markers) also occurred only in these double-mutated cells. Then, we corroborated that SPN-A566V increased the tumorigenic and stemness properties of the cells depending on p53 mutations using two p53-mutated breast cancer cell lines. In both cases, cells that overexpress SPN-A566V grew faster and formed larger tumors and showed a potentiation of the stemness properties. Our data confirm the relationship between SPN and p53 inactivation and demonstrate that SPN mutation alone is not able to initiate tumorigenesis, but it is a late event that promotes tumor progression and aggressiveness by increasing stemness and the pool of CD44+ CD24- cells.

SPN is one of the PP1 regulatory proteins involved in the dephosphorylation of pRB 17,28 that controls the expression of cell cycle genes 36,37. SPN-A566V interacts strongly with PP1α and PP1γ, but not with PP1β, as previously described for wild-type SPN 35. However, its homologous Neurabin-1, which is expressed exclusively in the neural tissue, binds PP1β 1,35,52,53, which means that SPN and Neurabin-1 have independent functions 1,35,53. The three isoforms of the catalytic subunit of PP1 form holoenzymes with different PP1 regulatory proteins and show different activities during the cell cycle 54-56: PP1α is the main isoform during G1 and the G1/S transition 42, while PP1β acts preferably in mitosis 54-58. Additionally, SPN-A566V induced a change in the localization of PP1. SPN is located preferentially in the cytoplasm and in the plasma membrane, but some studies suggest that it could also be expressed in the nucleus 1,59, whereas PP1α/γ are located mainly in the nucleus. When cells overexpress the SPN-A566V mutant, SPN and PP1α/γ colocalize in the cytoplasm and partially in the nucleus, suggesting that SPN-A566V directs PP1 to the cytoplasm, preventing its translocation to the nucleus and, therefore, partially preventing its activity on the P-pRB substrate. We do not fully understand the nature of the mislocalization. It may be a specific effect of the A566V mutation or an unwanted consequence due to the saturation of the transport system.

PP1 forms a complex with unphosphorylated or phosphorylated pRB through a PP1 regulatory protein 4,38-42. PP1 binds P-pRB from the end of mitosis to the middle of G1 4,60-62. We observed that SPN interacts specifically with both total and phosphorylated pRB in Ser807/811, two of the preferred PP1 dephosphorylation sites 43. SPN-A566V does not affect this interaction, but the holoenzyme PP1-SPN-A566V has a lower capacity to dephosphorylate P-pRB. PP1 interaction with other pocket proteins (p107 and p130) has not been described previously 55,63. In this work, we described for the first time that SPN is able to bind and dephosphorylate P-p107 Ser975 and P-p130 Ser672. SPN-A566V interacts weakly with both of them and the holoenzyme has lower ability to dephosphorylate P-p130 in Ser672. Although it could be possible that another phosphatase dephosphorylates P-p107 and P-p130 in other contexts, our data suggest that the PP1-SPN holoenzyme is not exclusive to P-pRB, but acts over all the pocket family proteins.

Pocket proteins collaborate in different phases of cell cycle regulation 44,64-66. We showed that SPN-A566V cells synchronized at G0 through serum deprivation recovered earlier than control cells by expressing higher levels of P-pRB, P-p107 and partially P-p130. Additionally, SPN-A566V cells synchronized at the end of G1 with mimosine also expressed higher levels of P-pRB and P-p130. However, the levels of phosphorylated pocket proteins remain constant after synchronization at the G2/M transition with nocodazole. As SPN-A566V cells have increased their proliferative capacity, our results suggest that they might progress faster through the cell cycle, with a shorter G1 phase and earlier S phase entry (Figure S4). The PP1-SPN holoenzyme seems to regulate the dephosphorylation of pocket proteins during the G0/G1 transition and at the end of G1 but do not act during the G2/M transition, which is in accordance with the fact that SPN binds exclusively PP1α/γ, but not PP1β, mainly involved in mitosis. PP1β could bind to a different PP1 regulatory protein at the exit of mitosis, but it remains unidentified 38-40. On the other hand, PNUTS (Phosphatase Nuclear Targeting Subunit) is a PP1 inhibitory protein with an important role in controlling PP1 activity during mitosis by inhibiting pRB dephosphorylation. However, PNUTS is only associated with a small proportion of PP1, so other proteins beyond PNUTS must regulate PP1 during the cell cycle, such as SPN 67. In fact, PNUTS and SPN bind PP1 in different regions without overlap 68. PNUTS is a context-dependent PP1 regulatory protein, and it is possible that the role of SPN in PP1 regulation and pocket protein dephosphorylation might be also dependent on the context, regarding either cell cycle or subcellular localization. Additionally, pRB could function as a substrate or as a PP1 regulatory protein since there are different pRB subpopulations performing different functions depending on the phosphorylation status 4. Different holoenzymes could be involved in the sequential control of pocket protein dephosphorylation during cell cycle progression, and each isoform/holoenzyme might have distinct specificity to different phosphorylated residues, like CDKs/Cyclin complexes, so that an initial dephosphorylation would be necessary to induce a conformational change before any other holoenzyme gains access to different residues 62. Moreover, p107 and p130 could partially compensate the absence of pRB in cell cycle regulation. However, this redundancy is not complete since several physiological properties are not equivalently altered in the two cell lines (as presented in Figures 2G, H). It has been suggested that these distinct activities may be dependent on the different E2F proteins regulated by the several pocket proteins. Interestingly, it has been reported that the effect on reprogramming was observed only for pRB, but not for other pocket proteins 69-71. Therefore, the effect on limiting dedifferentiation may explain the differential effects between pRB and other pocket proteins.

pRB also plays an important role in stem cell biology 72-74. It maintains such a balance between stem cell renewal and differentiation that, when deregulated, tumorigenesis is favored 75-78. Embryonic stem cells have a shorter G1 phase due to a high and non-cyclical CDKs/Cyclin activity and the absence of CDK inhibitors so that pRB and p107 are constantly phosphorylated and inactive, allowing cell proliferation 72-74. Therefore, it might be a connection between cell cycle and stem cell biology. The inactivation of pRB by phosphorylation produces the loss of its binding ability to E2F transcription targets, which induces the transactivation of genes involved in both the cell cycle and the pluripotency of cells. Furthermore, hypo-phosphorylated pRB bound to E2Fs recruits histone deacetylases and dramatically silences gene transcription epigenetically 79. These reports suggest that the effect of pRB on stemness may depend on its ability to retain epigenetic control.

pRB has another important role in controlling pluripotency since its inhibition facilitates induced pluripotent stem cell (iPSC) formation via caspase-mediated cleavage 80. More recently, pRB was reported to be directly involved in the transcriptional regulation of the pluripotency genes OCT4 and SOX2 81. SPN-A566V cells grow faster and have high levels of P-pRB as well as NANOG, OCT4 and SOX2. When pRB is dephosphorylated and active, OCT4 and SOX2 promoters are inhibited 82; thus, P-pRB may promote OCT4/SOX2 expression in SPN-A566V cells, which in turn induce NANOG 83,84. OCT4 regulates the self-renewal and differentiation of embryonic stem cells, at the same time controlling the cell cycle by increasing CDKs/Cyclin levels during the G1 phase and by preventing pRB dephosphorylation by PP1 82,85,86. However, further studies are needed to clarify if the PP1-SPN holoenzyme plays any role in the OCT4/pRB self-regulatory circuit. Although p107 and p130 also regulate stem cell biology, they might be involved in different functions than those of pRB; alternatively, the absence of pRB may be only partially compensated by the other pocket proteins.

## Conclusions

Our work has identified an oncogenic mutation of SPN, A566V, which affects both PP1-SPN interaction and PP1 phosphatase activity, especially over the pocket proteins. We also propose a connection between cell cycle and stem cell biology via SPN/PP1/pocket proteins. SPN-A566V cells have high levels of P-pRB, P-p107 and partially P-p130 during G0/G1 transition and at the end of G1. Thus, the G1 phase would be shorter, and cells would proliferate more rapidly and express some CSC markers, making them more aggressive. In conclusion, SPN-A566V would be an event that promotes p53-depending tumorigenesis by inducing the CSC pool in breast tumors. Indeed, SPN might have a predictive and prognostic value not only in breast cancer but also in any type of cancer.
