# Mutation of SPINOPHILIN (PPP1R9B) found in human tumors promotes the tumorigenic and stemness properties of cells: Erratum

**DOI:** 10.7150/thno.131824

**Published:** 2026-02-12

**Authors:** Eva M Verdugo-Sivianes, Ana M Rojas, Sandra Muñoz-Galván, Daniel Otero-Albiol, Amancio Carnero

**Affiliations:** 1 Instituto de Biomedicina de Sevilla, IBIS, Hospital Universitario Virgen del Rocio, Consejo Superior de Investigaciones Científicas, Universidad de Sevilla, Avda. Manuel Siurot s/n, 41013 Seville, Spain.; 2 CIBERONC, Instituto de Salud Carlos III, 28029 Madrid, Spain.; 3 Centro Andaluz de Biología del Desarrollo (CABD), CSIC-Universidad Pablo de Olavide, Sevilla, Spain.

In the originally published version of this paper, Figure 5, panel C, there is an erroneous WB in the *cell lysate* part of the figure. We performed this experiment several times, always obtaining the same result. At some point, the Western blots must have been confused, and an incorrect image was included.

To correct this mistake, we are providing a new correct WB and new Figure 5C, which shows that the result is unchanged. In addition, we are submitting the uncropped Western blots (supplementary figure 1) for verification.

We sincerely apologize for this error.

## Figures and Tables

**Figure A:**
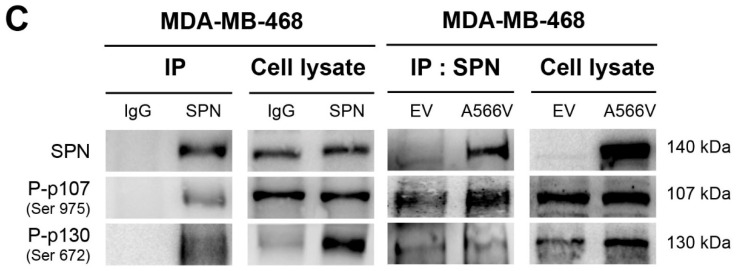
Corrected Figure 5C.

