# Supplementary material for: Mutation of SPINOPHILIN (PPP1R9B) found in human tumors promotes the tumorigenic and stemness properties of cells
Source: Theranostics. 2021 Jan 19;11(7):3452–71. doi: 10.7150/thno.53572 (PMC7847670; doi:10.7150/thno.53572)
Supplement: Supplementary file 1 — Supplementary figures and tables. [file thnov11p3452s1.pdf]

SUPPLEMENTARY INFORMATION

Mutation of SPINOPHILIN (PPP1R9B) found in human tumors promotes the tumorigenic and stemness properties of the cells

Authors: Eva M Verdugo-Sivianes[1,2], Ana M Rojas[3], Sandra Muñoz-Galván[1,2], Daniel Otero-Albiol[1,2] and Amancio Carnero[1,2*]

[1]Instituto de Biomedicina de Sevilla, IBIS, Hospital Universitario Virgen del Rocio, Consejo Superior de Investigaciones Científicas, Universidad de Sevilla, Avda. Manuel Siurot s/n, 41013 Seville, Spain.

[2]CIBERONC, Instituto de Salud Carlos III, 28029 Madrid, Spain.

[3]Centro Andaluz de Biología del Desarrollo (CABD), CSIC-Universidad Pablo de Olavide, Sevilla, Spain.

***Corresponding author:** Amancio Carnero

Address: Instituto de Biomedicina de Sevilla (IBiS), Hospital Universitario Virgen del Rocio, CSIC, Universidad de Sevilla, 41013 Seville, Spain. acarnero-ibis@us.es

Supplementary Table S1: Mutations in SPN protein found in human tumors

Supplementary Table S2: Correlation between SPN mutations, p53 mutations and other relevant inactivating mutations of the p53 pathway

Supplementary Figure S1: Mutations in the PP1 interaction region of SPN protein.

Supplementary Figure S2: Selection of SPN-A566V mutation.

Supplementary Figure S3. Molecular characterization of breast cancer cell lines.

Supplementary Figure S4: Cells with the SPN-A566V mutation cycle faster and proliferate more than control cells.

Supplementary Figure S5: The holoenzyme PP1-SPN-A566V showed reduced ability to dephoshorylate pRB.

Table S1: Mutations in SPN protein found in human tumors

Sample (ID)	Study	AA change	Type of mutation	Copy	Allelic freq
TCGA-ER-A19T-01	Cutaneous melanoma (TCGA)	G421V	Missense		0.12
TCGA-BQ-7058-01	Papillary renal cell carcinoma (TCGA)	Y425Lfs *58	FS ins	Gain	0.27
TCGA-VS-A9UI-01	Cervical squamous cell carcinoma (TCGA)	E428Q	Missense	Deletion parcial	0.11
nsclc_mskcc_2018s 47	Non-Small Cell Lung Cancer (MSK 2018)	G430R	Missense		0.06
TCGA-56-1622-01	Lung squamous cell carcinoma (TCGA 2012)	P435S	Missense	Gain	0.26
TCGA-EE-A2GC-06	Cutaneous melanoma (TCGA)	E440K	Missense	Amplifi ca-tion	0.69
NCIH2291_LUNG	Mixed Cancer Types (Broad 2019)	D442A	Missense	Diploid	
BP-014 T	Lung tumor biopsy (HUVR, our mutational analysis 2014)	P456	Silent		
coadread_dfci_2016 _1230	Colorectal adenocarcinoma (DFCI 2016)	X458_ splice	*Splice*		
MEL-IPI_Pat117-Tumor-SM-5X2QU	Melanoma (Van Allen 2018)	Y462H	Missense		0.35
coadread_dfci_2016 _251	Colorectal adenocarcinoma (DFCI 2016)	E465K	Missense		
TCGA-A5-A0G2-01	Carcinoma seroso papilar uterino (TCGA)	R469C	Missense	Diploid	0.27
MEL-IPI_Pat21-Tumor-SM-4DK1H	Cutaneous melanoma (Van Allen 2018)	R469C	Missense		0.07
NCIH1694_LUNG	Mixed Cancer Types (Broad 2019)	A478P	Missense	Diploid	
TCGA-IR-A3LK-01	Cervical squamous cell carcinoma (TCGA)	S480C	Missense	Diploid	0.41
PGM36	Esophagogastric adenocarcinoma (TMUCIH 2015)	R488H	Missense		
NCIH1155_LUNG	Mixed Cancer Types (Broad 2019)	R488H	Missense	Diploid	
TCGA-AP-A0LD-01	Uterine endometrioid carcinoma (TCGA)	R488H	Missense	Diploid	0.75
coadread_dfci_2016 _3683	Colorectal adenocarcinoma (DFCI 2016)	E490K	Missense		
DMS454_LUNG	Mixed Cancer Types (Broad 2019)	E493K	Missense	Amplifi cation	
HEC59_ENDOMET RIUM	Mixed Cancer Types (Broad 2019)	E500V	Missense	Diploid	
CSCC-38-T	Cutaneous squamous cell carcinoma (MD Anderson 2014)	S503F	Missense		0.18
5-PT035-T1	Skin cancer, non-melanoma (UNIGE 2016)	I508T	Missense		0.33
MHHCALL3_HAEM ATOPOIETIC_AND_ LYMPHOID_TISSU E	Mixed Cancer Types (Broad 2019)	G512S	Missense	Diploid	
TCGA-EJ-7125-01	Prostate adenocarcinoma (TCGA 2015)	M513I	Missense	Diploid	0.06
TCGA-J8-A3O2-01	Papillary thyroid cancer (TCGA)	G516R	Missense	Diploid	0.2
coadread_dfci_2016 _3640	Colorectal adenocarcinoma (DFCI 2016)	A517V	Missense		
TCGA-Q1-A73O-01	Cervical squamous cell carcinoma (TCGA)	D518H	Missense	Diploid	0.17
SNU1_STOMACH	Mixed Cancer Types (Broad 2019)	M519T	Missense	Diploid	
TCGA-B1-A654-01	Papillary renal cell carcinoma (TCGA)	M519T	Missense	Gain	0.6
OVISE_OVARY	Mixed Cancer Types (Broad 2019)	V531M	Missense	Diploid	
coadread_dfci_2016 _1230	Colorectal adenocarcinoma (DFCI 2016)	V531M	Missense		
TCGA-ER-A19H-06	Cutaneous melanoma (TCGA)	R539L	Missense	Diploid	0.08
HS695T_SKIN	Mixed Cancer Types (Broad 2019)	R539W	Missense	Amp	
TCGA-IB-7651-01	Pancreatic adenocarcinoma (TCGA)	T555P	Missense	Diploid	0.26
CHC892T	Hepatocellular carcinoma (Inserm 2015)	V558M	Missense		
coadread_dfci_2016 _3024	Colorectal adenocarcinoma (DFCI 2016)	T561A	Missense		
TCGA-37-3789-01	Lung squamous cell carcinoma (TCGA)	Q562K	Missense	Diploid	0.17
TCGA-AO-A128-01	Breast invasive ductal carcinoma (TCGA 2015)	A566V	Missense	Diploid	0.29

BT-053 T	Lung tumor biopsy (HUVR, our mutational analysis 2014)	A566V	Missense		
TCGA-HU-A4GU-01	Stomach adenocarcinoma (TCGA 2014)	R570L	Missense	Diploid	0.31
5-PT049-T1	Skin cancer, non-melanoma (UNIGE 2016)	T572I	Missense		0.33
TCGA-B5-A11E-01	Uterine endometrioid carcinoma (TCGA)	R575*	Nonsense	Diploid	0.33
RP-1066_PCProject_EDCDTqcM_T1_v1_Exome_OnPrem	Prostate Adenocarcinoma (The Metastatic Prostate Cancer Project 2019)	R577W	Missense	Diploid	0.1
TCGA-D1-A177-01	Uterine endometrioid carcinoma (TCGA)	R582Q	Missense	Diploid	0.46

Data obtained from both the cBioPortal database and our own mutational analysis (updated Sept 2019). AA = amino acid; freq. = frequency; Splice = change in alternative splicing; FS ins = insertion with change of reading frame.

Table S2: Correlation between SPN mutations, p53 mutations and other relevant inactivating mutations of the p53 pathway

SPN mutation	p53 mutation	Other mutations
G421V	No	SMAD3 (SMAD3-LRP5), BRCA2 (P1496T)
Y425Lfs *58	No	FOXO1 (S386fs*58)
E428Q	No	PIK3CA (E545K)
G430R	X261_splice	No
P435S	R337L	PIK3CA (Amp)
E440K	P177L, D1743Y, D1743A, P1702S	CDKN2A (Q50*)
D442A	G154V	No
P456	Mutated p53	Not determined
X458_ splice	No	NOTCH1 (T432M), SMAD3 (R287W), WNT4 (A193V)
Y462H	No	NOTCH4 (Q861*), WNT8A (G107D), MAPK4,7 mut
E465K	No	No
R469C	S241F, R342*, R379C	MDM2 (R169I, S259Y), NOTCH1,3,4 mut
R469C	No	B-CAT (P639S), MAPK4, 8 mut
A478P	X261_splice	NOTCH4 (C133Lfs*20)
S480C	E285K	NOTCH3 (*2322Sext*106)
R488H	C124Lfs*25	No
R488H	A142V, Y205F, R273H	NOTCH4 (A162T)
R488H	No	PIK3CA (R38H, R108H), PTEN (E43*, F238Vfs*5)
E490K	V143Afs*5	No
E493K	V157F	NOTCH2,4 mut
E500V	S95F, R273H	NOTCH1,3 mut
S503F	R248W	NOTCH1,2,3 mut
I508T	R213*, R342*, P420L	NOTCH2 (T1360I, R1372W)
G512S	No	CDKN2A (DeepDel 9p21.3)
M513I	G1513W	NOTCH4 (X1734_splice)
G516R	No	No
A517V	No	NOTCH2,3 mut
D518H	No	MAPK9 (Q293E), pRB (P374A), PIK3CA (Amp)
M519T	No	NOTCH4 (R801H), BRCA2 (S309Hfs*15), MAPK14 (E253A, L289F)
M519T	No	BRCA2 (K3313T)
V531M	No	MDM2 (Amp), NOTCH1 (S1027*), PIK3CA (C420R)
V531M	No	NOTCH1 (T432M), WNT4 (A193V)
R539L	No	CDKN2A (X153_splice), NOTCH1,3 mut
R539W	No	Wnt2,6 (Amp), MAPK8,10 Deepdel
T555P	No	MDM2 (S218I), B-CAT multiple mutations, NOTCH1,3 mut
V558M	No	WNT multiple mutations, BRCA2 (G1376R, E2123K), NOTCH3,4 mut
T561A	No	WNT16 (G167Afs*17), MAPK12 (X143_splice)
Q562K	R65*	NOTCH1 (E2115*)
A566V	R342*	PTEN (R130*), WNT2B (R253C)
A566V	Mutated p53	Not determined
R570L	P191del	NOTCH2,3 mut
T572I	A159V, R213*	NOTCH1 (W287*)
R575*	R342*	NOTCH2 (P522H, E910D)
R577W	No	BRCA2 (G602Kfs*13), MAPK3,9 (Amp)
R582Q	No	PTEN (E157), NOTCH2,3

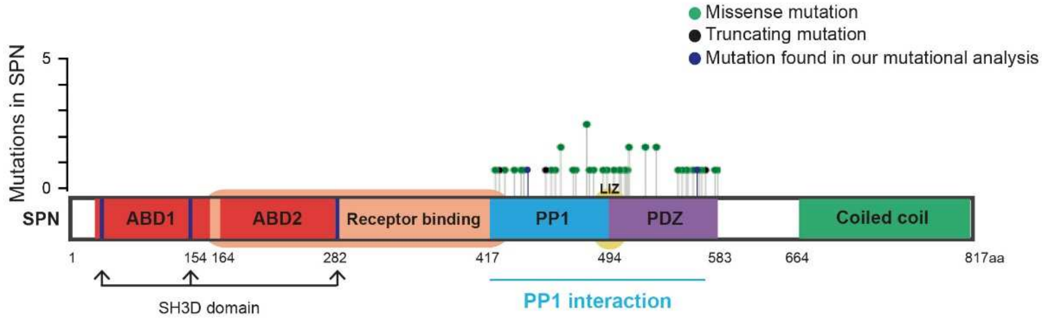

Figure S1: Mutations in the PP1 interaction region of SPN protein. Representation of the mutations of SPN found in the PP1 interaction region in human tumors, including both mutational analysis (cBioportal database and our own mutational analysis).

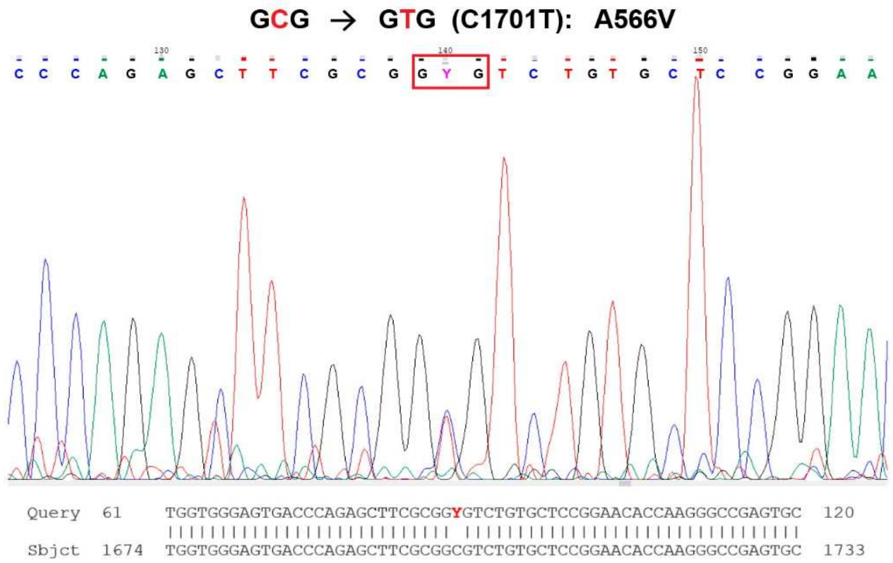

Figure S2: Selection of SPN-A566V mutation. Sequecing result of the mutation SPN-A566V found in our own mutational analysis.

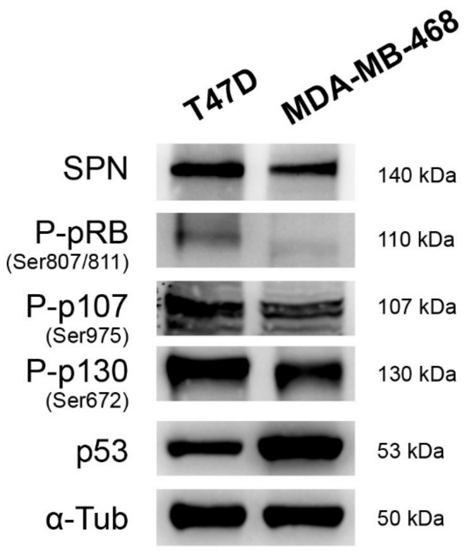

Figure S3. Molecular characterization of breast cancer cell lines. Measurement of the levels of SPN, P-pRB (Ser807/811), P-p107 (Ser975), P-p130 (Ser672), p53 and α-Tubulin in the two breast cancer cell lines used, T47D and MDA-MB-468, by western blot.

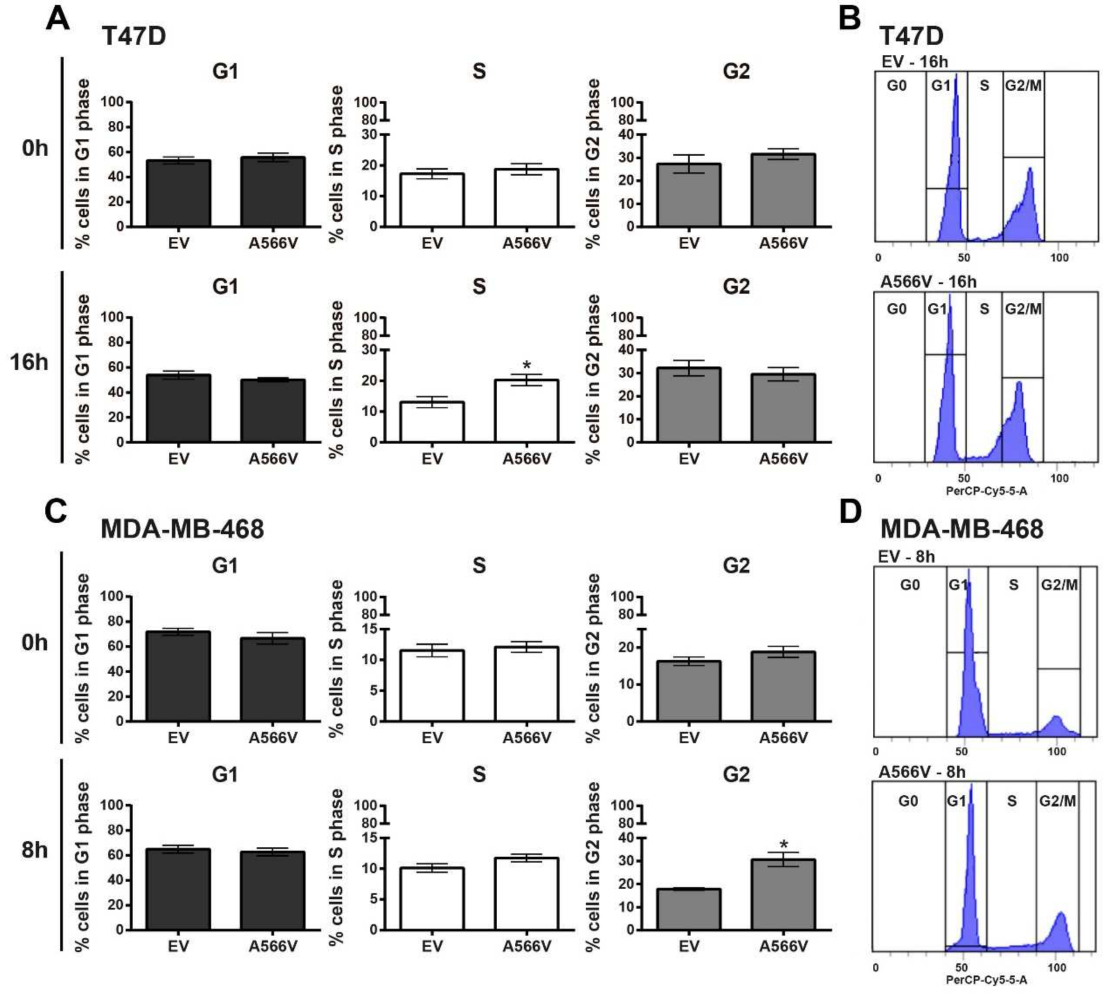

Figure S4: Cells with the SPN-A566V mutation cycle faster and proliferate more than control cells. A-C) Measurement by FACS the percentage of cells in each phase of the cell cycle in T47D and MDA-MB-468 control and SPN-A566V cell lines after serum deprivation for 24 h. **B-D)** Representative images of the cell cycle experiments. The mean of a minimum of 3 independent experiments performed in triplicate ± standard deviation is represented. Statistical analysis was performed with the t-Student test * $p < 0.05$.

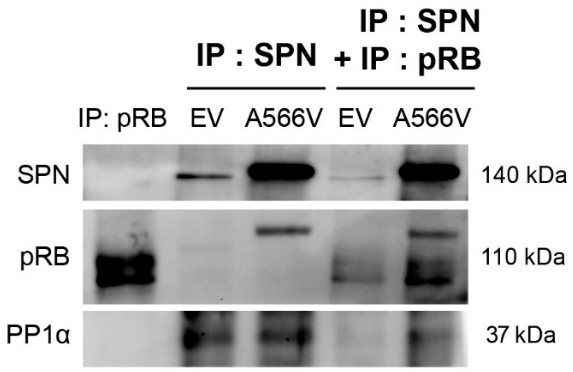

Figure S5: The holoenzyme PP1-SPN-A566V showed reduced ability to dephoshorylate pRB. Lanes 1-3: Co-immunoprecipitation of total pRB, PP1α and SPN in nontransfected parental HEK-293T cells (lane 1), HEK-293T transiently transfected with the empty vector (lane 2) or with the SPN-A566V mutant (lane 3). Protein extracts were subjected to immunoprecipitation with anti-pRB (lane 1) or anti-SPN (lanes 2-3), and the immunoprecipitates were analyzed with anti-SPN, anti-PP1α, anti-pRB antibodies. Lanes 4-5: Phosphatase assay of the PP1-SPN holoenzyme. SPN was immunoprecipitated in HEK-293T transiently transfected with the empty vector (lane 4) or with the SPN-A566V mutant (lane 5). At the same time, total pRB was immunoprecipitated in the nontransfected parental HEK-293T cells to be used as a substrate over 40 min. The results were analyzed by western blot analysis. A representative image of 3 experiments performed independently is shown.
